# Connecting Anxiety and Genomic Copy Number Variation: A Genome-Wide Analysis in CD-1 Mice

**DOI:** 10.1371/journal.pone.0128465

**Published:** 2015-05-26

**Authors:** Julia Brenndörfer, André Altmann, Regina Widner-Andrä, Benno Pütz, Darina Czamara, Erik Tilch, Tony Kam-Thong, Peter Weber, Monika Rex-Haffner, Thomas Bettecken, Andrea Bultmann, Bertram Müller-Myhsok, Elisabeth E. Binder, Rainer Landgraf, Ludwig Czibere

**Affiliations:** 1 Department of Behavioral Neuroendocrinology, Max Planck Institute of Psychiatry, Munich, Germany; 2 Department of Statistical Genetics, Max Planck Institute of Psychiatry, Munich, Germany; 3 Department of Molecular Genetics of Affective Disorders, Max Planck Institute of Psychiatry, Munich, Germany; 4 Institute of Human Genetics, Helmholtz Zentrum München, Munich, Germany; 5 Institute of Human Genetics, Technische Universität München, Munich, Germany; University of Illinois at Chicago, UNITED STATES

## Abstract

Genomic copy number variants (CNVs) have been implicated in multiple psychiatric disorders, but not much is known about their influence on anxiety disorders specifically. Using next-generation sequencing (NGS) and two additional array-based genotyping approaches, we detected CNVs in a mouse model consisting of two inbred mouse lines showing high (HAB) and low (LAB) anxiety-related behavior, respectively. An influence of CNVs on gene expression in the central (CeA) and basolateral (BLA) amygdala, paraventricular nucleus (PVN), and cingulate cortex (Cg) was shown by a two-proportion *Z*-test (*p* = 1.6 x 10^-31^), with a positive correlation in the CeA (*p* = 0.0062), PVN (*p* = 0.0046) and Cg (*p* = 0.0114), indicating a contribution of CNVs to the genetic predisposition to trait anxiety in the specific context of HAB/LAB mice. In order to confirm anxiety-relevant CNVs and corresponding genes in a second mouse model, we further examined CD-1 outbred mice. We revealed the distribution of CNVs by genotyping 64 CD 1 individuals using a high-density genotyping array (Jackson Laboratory). 78 genes within those CNVs were identified to show nominally significant association (48 genes), or a statistical trend in their association (30 genes) with the time animals spent on the open arms of the elevated plus-maze (EPM). Fifteen of them were considered promising candidate genes of anxiety-related behavior as we could show a significant overlap (permutation test, *p* = 0.0051) with genes within HAB/LAB CNVs. Thus, here we provide what is to our knowledge the first extensive catalogue of CNVs in CD-1 mice and potential corresponding candidate genes linked to anxiety-related behavior in mice.

## Introduction

With the advances in genome-wide screening arrays and sequencing technologies, scientists were enabled to examine genetic variations and their effect on behavioral phenotypes. In recent years a new type of variation became increasingly important: the copy number variants (CNVs). Not only have CNVs already been associated with common disorders and metabolic diseases like asthma, type 2 diabetes, obesity and cancer [1–4], they also have been reported to affect disease susceptibility of neurological disorders including Parkinson’s disease, Alzheimer’s disease, autism, schizophrenia, bipolar disorders and anxiety disorders [5–17]. Further, a large and common CNV in mice including the Glyoxalase 1 (*Glo1*) locus has been associated with anxiety-like behavior [18]. Although the general impact of CNVs on (disease) phenotypes is not clear yet, there is evidence from many distinct studies pointing to their involvement in phenotypic expression. Thus, considering the mechanisms by which CNVs might act on gene expression and their high abundance across the genome, their contribution is likely to be of importance [19].

There are multiple potential mechanisms that explain how CNVs might contribute to distinct diseases and behavioral phenotypes such as anxiety-related behavior. For instance, a direct change of gene dosage following copy number alterations [20] is an obvious mode of action. More complex, however, are effects mediated by changes in copy numbers of enhancers and repressors, which were shown to act as *cis*-regulatory domains even though they extend long distances outside the coding region itself [21]. Furthermore, it is conceivable that CNVs mediate their effects by physically impairing the access of genes to the transcription machinery or by influencing transvection [22–24]. Hence, theoretically, CNVs could regulate the transcription of genes beyond their breakpoints. Although different detection methods exist and many studies have been performed so far, the detection of CNVs and the analysis of their effects still remain challenging.

Animal models such as the inbred HAB/LAB (high/low anxiety-related behavior) mouse model [25–27] represent an ideal tool for revealing the complex impact of CNVs on behavioral phenotypes. In order to provide a large-scale analysis of CNVs influencing anxiety-related behavior, we decided to not only apply three different detection methods to screen for CNVs in the HAB/LAB mouse model, but also to analyze CNVs in the genomic context of a second mouse model (CD-1 outbred mice). Thus, here we provide, first, an extensive study of anxiety-relevant CNVs and corresponding genes and, second, a catalogue of CNVs in CD-1 mice that might serve as basis for subsequent studies on the effects of CNVs.

## Material and Methods

### Ethics statement and general remarks

All animal experiments were conducted in accordance with the current regulations for animal experimentation in Germany and the European Union (Council of the European Communities Directive 86/609/EEC) and were approved by the Government of Upper Bavaria.

Information on genomic positions all refer to the UCSC genome browser assembly mm9 (http://genome.ucsc.edu/) [28].

### Animals and behavioral experiments

All animals were housed in the animal facility of the Max Planck Institute of Psychiatry under standard conditions, i.e., a temperature of 23 ± 2°C, a relative air humidity of 60 ± 5% and a 12/12-hour light-dark cycle with beginning of light phase at 8 a.m. Animals were kept in groups of up to four animals per type II standard cage with nesting and bedding material, having access to food pellets (Altromin GmbH, Lage, Germany) and tap water *ad libitum*.

Male HAB and LAB mice used in this study were selected from generations 35 to 41, bred in the animal facility of the Max Planck Institute of Psychiatry (Munich, Germany). The HAB/LAB mouse model was described before by Krömer *et al*. [25]. Briefly, the HAB and LAB mouse lines were bred following bidirectional breeding protocols successfully applied in rats before [26, 29, 30]. Depending on their performance on the elevated plus-maze (EPM) [31], individuals of a population of more than 250 animals from over 25 litter of outbred Swiss CD-1 mice were chosen to either found the HAB or LAB mouse line. After nine generations of outbreeding across families but within behavioral restrictions, a strict inbreeding protocol was followed, resulting in two mouse lines showing a stable anxiety-related phenotype, with HAB mice spending less than 20% and LAB mice more than 50% of the test time on the open arm of the EPM.

**Fig 1:**
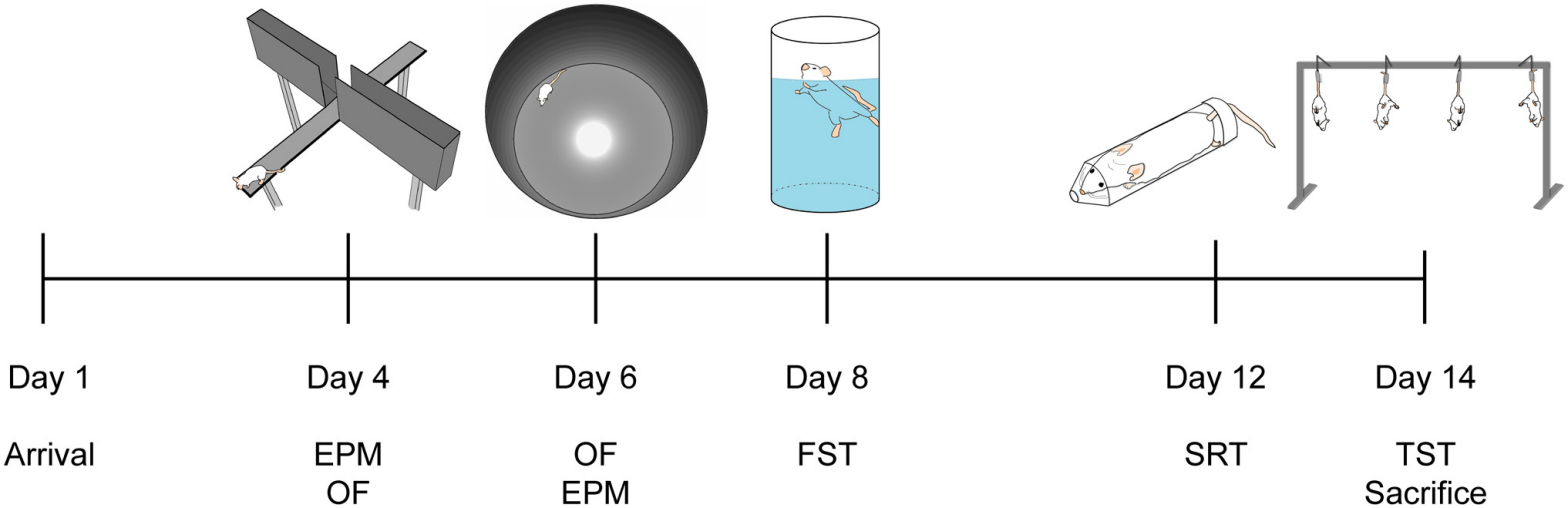
Test series to phenotypically characterize CD-1 mice. Each animal had to perform five tests (EPM, OF, FST, SRT, TST) in a row as shown. Animals tested on the EPM on day 4 were tested in the open field on day 6 and vice versa.

Male outbred Swiss CD-1 mice used in this study were purchased from Charles River (Sulzfeld, Germany) and delivered at the age of eight weeks in eight different batches of 48 animals each (in total 384 animals). Deliveries of discrete batches were made at an interval of at least one week. The CD-1 mice of each batch were phenotypically characterized in a series of five tests covering different facets of anxiety (Fig 1): after arrival and a 4-day-habituation, half of the animals were tested on the EPM [31], the other half in the open field (OF) [32], and two days later vice versa. On day eight after arrival, the forced-swim test (FST) [33] was performed, followed by stress-reactivity test (SRT) [34] and tail suspension test (TST) [35] on day 12 and 14, respectively. Behavioral tests were conducted between 9 a.m. and 1 p.m. under standard housing conditions. EPM and OF were analyzed by means of the tracking software Any-maze v4.72 (Stoelting, Wood Dale, IL, USA). Other tests were recorded and analyzed by experienced researchers using Eventlog v1.0.

EPM: The apparatus we used was made of grey polyvinylchloride (PVC). It consisted of two open arms (30 × 5 cm, 300 lx) and two closed arms (30 × 5 × 15 cm, 10 lx) extending from a central platform (5 × 5 cm) and elevated by four legs (40 cm). Each mouse was placed in the central zone facing a closed arm. Its behavior was recorded for 5 min.

OF: The test apparatus consisted of a round PVC wall of 40 cm height, framing a field of 60 cm in diameter. Intensity of light differed between brighter inner zone and darker outer zone of about 15 lx. Each mouse was placed in the inner zone.

FST: Mice were placed in a 2 l glass beaker filled with 1.75 l tap water (23°C). Behavior was recorded for 6 min and analyzed with a customized Eventlog program, differentiating between freezing, floating, struggling and swimming. Floating was defined as not showing any movements except very slight balancing movements.

SRT: The increase of corticosterone (Cort) levels in the blood was detected after a 15-minute period of restrained stress, where animals were placed into a 50 ml plastic tube (11.4 x 2.8 cm²) with holes for ventilation. Cort levels were measured by radioimmunoassay.

TST: Four mice were suspended in parallel from a metal frame (height: 37 cm) by fixing their tail tips with an adhesive tape. Behavior was videotaped for 6 min and analyzed for immobility (no movement at all) and struggling, using a customized Eventlog program.

### DNA extraction

For DNA extraction from tail tips of both HAB/LAB and CD-1 mice, a NucleoSpin Tissue kit (Macherey-Nagel, Düren, Germany) was used, following the manufacturer’s instructions provided in the “standard protocol for human and animal tissue and cultured cells”. In case of NGS only, the DNA was extracted using a DNeasy blood & tissue kit (Qiagen, Hilden, Germany), following the guideline provided with the kit. DNA concentration was measured on a NanoPhotometer (Implen, München, Germany) and DNA quality was checked by gel electrophoresis using 1.0% agarose gels (or 0.7% for NGS samples).

### CNV detection…

We screened the genome of HAB/LAB mice for CNVs using three different detection methods, (1) array comparative genomic hybridization (aCGH), (2) the Jax Mouse Diversity Genotyping Array (JaxMDGA), and (3) next-generation sequencing (NGS), as described below.

#### … by aCGH

DNA extracted from tail tips of two 16-week-old male HAB and LAB mice each, and from brain tissue of only one pair of these mice, respectively, was sent to Roche NimbleGen (Madison, WI, USA) to access NimbleGen’s full CGH microarray service (Mouse CGH 3x720 K Whole-Genome Tiling Array; probes: 50- to 75-mers; median probe spacing: 3.5 kb). Briefly, according to NimbleGen’s supplied information sheet (NimbleGen CGH Services: Guide to Your CGH Data v3.0; 2009), the signal intensity was spatially corrected based on the X and Y coordinate position on the array using locally weighted polynomial regression. The Cy3 (HAB sample) and Cy5 (LAB sample) signal intensities were normalized to one another using qspline normalization. Roche NimbleGen applied the segMNT algorithm that identifies copy number changes using dynamic programming to globally minimize the sum of squares error relative to the segment means. Assessing the provided data, we defined the final set of high confidence CNV calls if segMNT-defined segments had a mean log_2_ signal ratio (Cy3 signal/Cy5 signal) greater/less than +/-0.09 in all three assays.

#### … by JaxMDGA

The high-density Jax Mouse Diversity Genotyping Array (The Jackson Laboratory, Bar Harbor, ME, USA), containing 623,124 SNP and 916,269 invariant genomic probes [36], was applied to screen the genome of HAB/LAB and CD-1 mice for CNVs. We accessed the basic service offered by The Jackson Laboratory, including DNA extraction, sample preparation, array hybridization and provision of raw data. We provided tail tips of one male HAB and LAB mouse each, and of 64 male CD-1 mice, respectively, to analyze their genome on a high-density Jax Mouse Diversity Genotyping Array (The Jackson Laboratory) [36]. The CD-1 mice, a subgroup of the 384 animals described in Materials and Methods, were chosen based on their behavior averaged over all tests, with 24 mice showing highest, 24 lowest, and 16 intermediate anxiety-related behavior (for details on selection criteria see reference [37]). The array was performed in two batches of 32 samples each.

With LAB defined as reference sample, we performed CNV calling in the raw data of HAB/LAB mice by applying the Hidden Markov Model-based function “simple CNV” implemented in the “MouseDivGeno” R package [38]. This function integrates normalized intensities from SNPs and exons. To infer the most likely state from three possible states (loss, normal, or gain compared to the reference sample) the function uses “HiddenMarkov”, an existing HMM R package. [39]

CNV calling in the raw data of CD-1 mice was performed by applying the “simple CNV” function for each pair of animals, i.e. each animal was once declared as reference sample. Thus, we increased the sensitivity for CNV calls. By contrast, a selection of just one single sample as reference would lead to a loss of information about potential CNVs (for a detailed explanation see S1 Text). However, discrepancies in breakpoint definition between different pairs of animals could occur. We solved this problem by unifying the breakpoints of respective CNVs, i.e. we assessed the starting and end points in a way the CNV was defined as large as possible (see S1 Fig). By means of the “normalizeForSimpleCNV” function, a subfunction of the “simple CNV” function, we were able to calculate the mean normalized intensities of all probes within those unified CNVs for each sample separately. These intensity values were required for the association study described below.

#### … by NGS

The DNA of six male HAB and LAB mice each was pooled to form a single HAB and LAB sample, respectively, to be sequenced on a SOLiD 4 System (Applied Biosystems, Foster City, CA, USA). After shearing the genomic DNA to an average size of 2,000 bp using a Covaris S2 system (Covaris, Woburn, MA, USA), 2x60 bp mate-paired libraries were prepared following the Applied Biosystems’ Mate-Paired Library Preparation guide (part # 4460958 Rev. A, revision date: March 2011). A quality control of libraries was performed using an Agilent 2100 Bioanalyzer (Agilent Technologies, Böblingen, Germany) and quantification was done by qPCR using a SOLiD Library TaqMan Quantification kit (Applied Biosystems, Cat. # A12127). For both libraries a 2 x E80 bead preparation scale was chosen, using an input of 1 pM each. Each E80 preparation was then loaded onto one full slide and sequencing was performed in two runs with one HAB and one LAB library at a time. Subsequent analysis of sequencing data was done as follows:

In a first step all reads with an average quality score (Phred-like score) below 10 were removed. For aligning the paired sequences to the mouse reference genome (UCSC genome browser version mm9) the two burrows-wheeler aligners bowtie (v0.12.7) [40] and BWA (v0.5.7) [41] were used in a step-wise procedure. First, bowtie was used to align read pairs in correct orientation (i.e., expected orientation and distance) to the reference genome. Second, the slower but more flexible BWA software was used for aligning previously unmapped read pairs in alternative orientations (i.e., unexpected orientations and distances). Third, BWA was used to align single reads (i.e., reads where the mate did not pass the quality control). The rationale behind this stepwise procedure was to leverage the computational performance of bowtie as well as the flexibility of BWA. Allowing reads to be aligned in alternative orientations and distances is crucial for the detection of large structural variants, since large deletions and insertions cause an increase and a reduction, respectively, in the distance between the mates, while inversions are detectable with unexpected orientations of the two mates (see Xi et al. [42] for a review). The alignments were sorted according to genomic locations and successfully mapped reads from the three steps above were merged into a single alignment file. For post-processing we used the tools Picard (http://picard.sourceforge.net/) and SAMtools [43].

CNV calling was based on a depth-of-coverage (DOC) approach [42]. Briefly, this approach works by comparing the effective coverage in one region in the LAB sample to the effective coverage in the same region in the HAB sample: if, for instance, in HAB this region shows four-times the coverage compared to the LAB sample, then this particular region is likely to be a CNV with four-times the copies. The CNV calling was performed using our newly developed software CNVfinder (http://cnvfinder.sourceforge.net/). Briefly, CNVfinder divides the genome into equally sized bins. Next, the coverage for each bin is computed as the sum of (i) the number of reads that are aligned to that bin and (ii) the number of read pairs whose insert size are covering that bin. This was done separately for the HAB and LAB samples. Finally, CNVfinder works at one chromosome at a time and compares the coverage for each bin on that chromosome in the two samples using Fisher’s exact test. The derived p-values are corrected for multiple testing using the method by Benjamini and Hochberg [44] resulting in a q-value for a false discovery rate. Candidate CNVs were identified by consecutive stretches of bins all exceeding a q-value threshold; candidate CNVs were extended to the left and right by including bins exceeding a lower (extension) q-value threshold.

For the analysis, we restricted the alignments to uniquely mapped mate pairs as indicated by a mapping score of 20 or better. Next, from the alignment, we extracted the information of chromosome, alignment position, insert size, and alignment flag (which contains information about the orientations of the two mates). Then, we removed PCR artifacts by removing all but one aligned read with the same start position and insert length. For the analysis with CNVfinder, the bin size was set to 200 bp, the initial CNV finding threshold was set to—log10(q) > 12 with a window size of 8 and the CNV extension threshold was set to—log10(q) > 10. Hence, the lower limit on the length for detectable CNV was 1,600 bp (8 x 200 bp). There were no limits regarding the maximal length of a CNV.

### Association analysis of CNV and behavior data

Prior to performing the association analysis of CNVs detected in 64 CD-1 mice (as described above) with all behavioral parameters tested, we aimed to take the relationship between those mice into account. Thus, behavioral traits were transformed with GenABEL's function "polygenic" and its GRAMMAR+ transformation output [45] in R. The association analysis was then performed based on a generalized linear model (i.e., the R function “glm”), using the transformed behavioral data. For measuring values of CNVs, the mean normalized intensities of JaxMDGA probes within the respective CNV were used. A likelihood-ratio test as implemented in the “anova” function of the “stats” R package was applied to calculate *p*-values. Distribution of *p*-values was checked by Q-Q plots (shown in S2 Fig). The *p*-values were adjusted for multiple testing using a correction method developed by Holm [46].

### Gene expression analysis

RNA extracted from basolateral (BLA) and central amygdala (CeA), hypothalamic paraventricular nucleus (PVN) and anterior part of cingulate cortex (Cg) of eight HAB and eight LAB animals was tested on a MouseWG-6 v1.1 Expression Bead Chip (Illumina, San Diego, CA, USA), containing 48,318 probes. The samples were not pooled (for details see reference [47]). A reanalysis of raw data from that earlier expression microarray experiment [47] was conducted (for raw date see GEO database GSE29015, http://www.ncbi.nlm.nih.gov/gds/). Data were normalized using the R function “vsn” [48]. Probes which were not sampled on at least one array were dropped and the remaining probes (N = 46,657) were further filtered by three criteria: 1. Probes had to map into genes with an EntrezGene-ID; 2. A unique alignment of probes to the genome (mm9) with a maximum of two mismatches was required; 3. Genes having a detection p-value greater than 1x10^-4^ were excluded. Probes passing the filtering conditions (N = 12,171) were used for subsequent analysis, using the R package “Limma” [49]. Significantly regulated genes were ranked using an empirical Bayes method [50]. Multiple testing was corrected for using the false discovery rate (FDR) approach [44]. Finally, significant expression differences were indicated by adjusted p-values less than 0.05, obtained from the performance of a contrast analysis.

We confirmed the results of the gene expression microarray by quantitative real-time PCR (qPCR). Brains of 11 HAB and 8 LAB males were cut in slices of 200 μm using a Microm HM560 cryostat (Microm, Walldorf, Germany). The brain areas of interest, CeA (bregma [51] 1.46 to 1.82), BLA (bregma 1.22 to 1.58), Cg (bregma 1.34 to 0.22), and PVN (bregma 0.58 to 0.94), were obtained by micropuncture, using sample corers of 0.5 mm (BLA, CeA) and 1.0 mm (Cg, PVN) in diameter (Fine Science Tools, Heidelberg, Germany). RNA was isolated by means of an RNeasy Plus Micro Kit (Qiagen) following the kit’s protocol. cDNA was transcribed from 0.5 μg RNA each, using a High Capacity cDNA Reverse Transcriptase Kit (Applied Biosystems), and further mixed with SYBR Green MasterMix (Qiagen) to be tested in duplicates on a LightCycler 480 (Roche Diagnostics, Mannheim; Germany). Primers (S1 Table) targeting two housekeeper and nine candidate genes shown to be both differentially expressed between HAB and LAB mice and part of functional protein association networks (STRING online software v9.0; http://string-db.org) were designed using the online tool Primer-Blast (www.ncbi.nlm.nih.gov/tools/primer-blast), and were purchased from Sigma-Aldrich (Taufkirchen, Germany). Data analysis was performed using the absolute quantification fit points method, provided with the LightCycler software. Sample data were analyzed relative to the housekeeper using the ΔΔCT-method [52], and normalized to the mean value of all HAB samples. Expression differences between HAB and LAB mice were determined applying the Mann-Whitney U test (SPSS software v16.0.1), assuming a significance threshold of 0.05.

### Correlation analysis of CNV and expression data

We tested the hypothesis that CNVs have an influence on gene expression using a two proportion *Z*-test, as described in S1 Text. Next, we examined the question of an underlying positive or negative correlation, starting with the creation of a list including all genes in copy number variable regions that were found to be differentially expressed between HAB and LAB mice in at least one of the tested brain regions (CeA, BLA, Cg, PVN). Information on copy number status was added by assigning a value of +1 (or -1) to all genes in regions of copy number gain (or loss, respectively) in HAB compared to LAB mice. The same was done for information on expression status, with a value of +1 (or -1) assigned to genes showing increased (or reduced, respectively) expression in HAB mice. Genes were assigned to both +1 and -1 when showing contradictory CNV status based on the three applied CNV detection methods, or contradictory expression status based on different microarray probes targeting the gene, respectively. We tested for a correlation between expression and CNV status by applying the Cohen’s weighted Kappa [53], using the “cohen.kappa” function of the “psych” library in R. The *p*-values were corrected for multiple testing using Holm’s correction method [46].

## Results

### CNVs related to anxiety

In total, we found 98 CNVs in HAB vs. LAB mice using aCGH, 180 and 5,851 CNVs by means of JaxMDGA and NGS, respectively. Their median size was calculated to be 2.4 kb (NGS), 8.9 kb (JaxMDGA) and 544.6 kb (aCGH). The size range of CNVs detected by the two arrays was in a range to be expected. [23, 54–58] All CNVs taken together, their total size of about 97.3 Mb (aCGH), 14.7 Mb (JaxMDGA) and 27.2 Mb (NGS) represent about 3.7% (aCGH), 0.6% (JaxMDGA) and 1.0% (NGS) of the whole genome, respectively. Sequences revealed by all three detection methods to differ in copy number covered about 4.8 Mb, which reflects 0.18% of the genome. In most cases, these findings were not contradictory, however, for about 168 kb altogether a copy number gain in HAB vs. LAB mice was shown by one method while a loss was discovered by the other methods, or vice versa. S2 Table details all CNVs detected by aCGH, JaxMDGA and NGS, including their genomic position and copy number status with respect to HAB mice. Further details on total and mean CNV size and overlap between results of distinct detection methods can be found in S3 Table. Examples of CNVs and their log2 signal intensity ratios (aCGH, JaxMDGA), or fold changes (NGS), respectively, are plotted in S3–S5 Figs.

**Fig 2:**
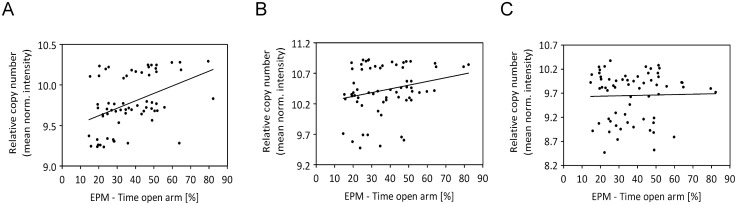
Association of copy number with anxiety-related behavior in CD-1 mice. Exemplarily, data of three associations resulting in nominal *p*-values reaching significance (*p* < 0.05), a trend (*p* < 0.1), and not reaching significance (*p* > 0.05), respectively, are shown. Each dot represents data of a single animal (N = 64). The relative copy number is represented by the mean normalized intensities of JaxMDGA probes within the respective CNV. **(A)** CNV no. 498; *P*_nom_ = 0.0009; regression line: y = 0.0091x + 9.4389. **(B)** CNV no. 164; *P*_nom_ = 0.0554; regression line: y = 0.0061x + 10.201. **(C)** CNV no. 453; *P*_nom_ = 0.9791; regression line: y = 0.0008x + 9.6225.

**Fig 3:**
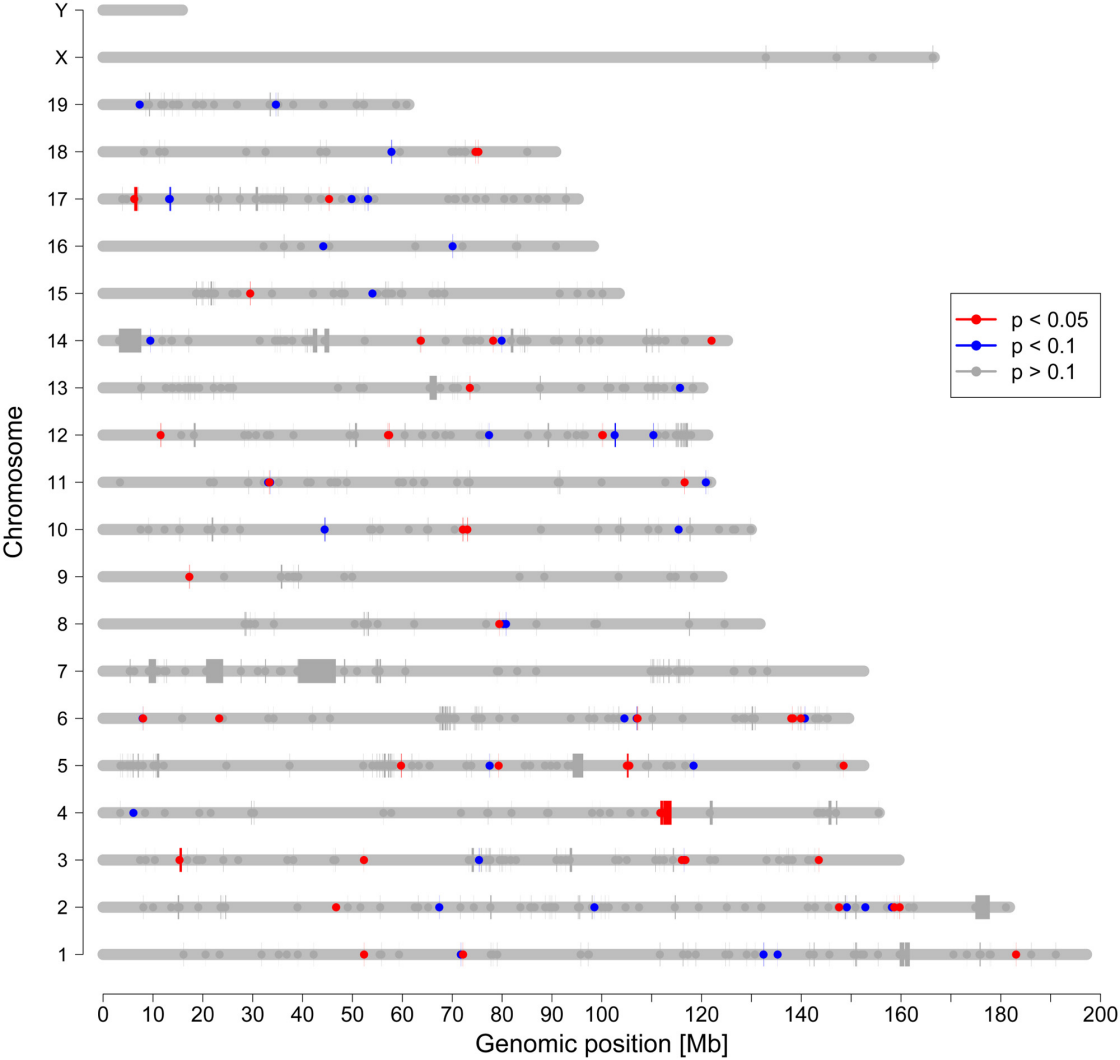
Distribution of CNVs in CD-1 mice. Chromosomes are indicated by grey horizontal lines. Start points of CNVs are marked by dots and lines are drawn to the end points. Due to limitations in resolution, a small CNV might appear as dot only. CNVs highlighted in blue or red were associated with anxiety-related behavior (time on the open arm of the EPM) with a nominal *p*-value less than 0.1 or 0.05, respectively.

With the intention to confirm the relevance of specific CNVs for anxiety-related behavior in a second mouse model, we genotyped 64 CD-1 mice using JaxMDGA. Comparing the raw data of all 64 animals to each other we revealed a total number of 764 CNVs with a median size of 14 kb. In order to define CNVs linked to anxiety-related behavior, we ran association analyses of CNVs with multiple behavioral parameters. Before correcting for multiple testing, we found 47 CNVs to be significantly associated (nominal *p*-value < 0.05) with the time the animals spent on the open arms of the EPM. For another 40 CNVs and the same behavioral parameter a trend (nominal *p*-value < 0.1) was shown. However, none of these effects survived the correction for multiple testing. The mean normalized intensities of all 764 CNVs and the time the 64 animals spent on the open arm of the EPM are shown in S4a Table. Data of three distinct CNVs are depicted exemplarily in Fig 2. In S4b and S4c Tables behavioral data of all tests are shown before and after GRAMMAR+ transformation, respectively. All CNVs including nominal and corrected *p*-values of the association analyses with all behavioral tests performed are outlined in S5 Table. Fig 3 illustrates the chromosomal distribution of CNVs found in CD-1 mice, with regions having nominal *p*-values less than 0.1 highlighted in color.

**Fig 4:**
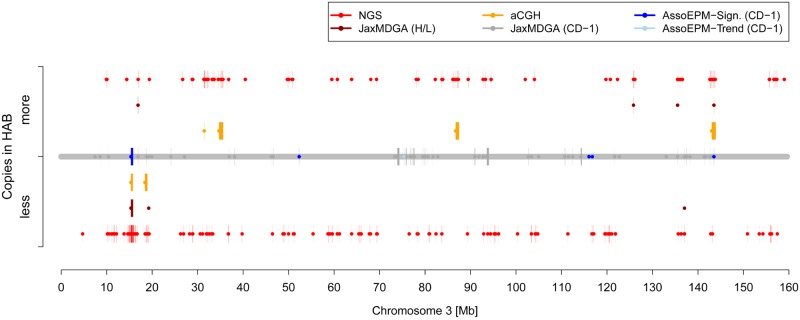
Genomic positions of CNVs on chromosome 3. The chromosome is indicated by a thick horizontal line (grey). Depending on the detection method, CNVs in HAB/LAB mice are depicted in orange (aCGH), dark red (JaxMDGA) and red (NGS), respectively. Data displayed above the grey line represent a copy number gain in HAB vs. LAB animals, data below a copy number loss. Data printed on the grey line show CNVs in 64 CD-1 mice, with those highlighted in color that could be associated with anxiety-related behavior (time on the open arm of EPM) with a nominal *p*-value less than 0.1 (light blue) or less than 0.05 (blue). Start points of CNVs are marked by dots and lines are drawn to the end points.

For the comparison of CNVs in CD-1 with those found in HAB/LAB mice, the distribution of CNVs in both mouse models is depicted in S6 Fig, and exemplarily for chromosome 3 in Fig 4.

### CNVs and gene expression

Out of 12,171 expression microarray probes that passed the filtering process, we found 291 probes in CeA, 117 in BLA, 297 in PVN, and 254 in Cg, respectively, to show expression differences between HAB and LAB mice. S6a Table details the probes’ genomic positions and respective results of microarray analysis. The 12,171 probes represented 8,981 protein coding genes of which 374 appeared to be differentially expressed in at least one of the tested brain regions. These 374 genes, as well as information on the microarray probes representing them, are listed in S6b Table. We confirmed the reliability of gene expression changes revealed by microarray analysis in a small subset of nine genes using qPCR. The data are shown in S7 Table.

We then statistically analyzed the overlap of genes that were tested in the expression microarray with CNVs which were confirmed by three detection methods, i.e. aCGH, JaxMDGA and NGS, by applying a two-proportion *Z*-test. All four brain regions individually (CeA, *p* = 5.9 x 10^-16^; BLA, *p* = 1.2 x 10^-21^; PVN, *p* = 3.0 x 10^-21^; Cg, *p* = 3.1 x 10^-33^) and all four brain regions taken together (*p* = 1.6 x 10^-31^) show highly significant *p*-values. Thus, we demonstrated that loci with variable copy number between HAB and LAB mice include significantly more differentially expressed genes than it is to be expected if CNVs would not influence expression levels. In other words, CNVs affect the expression level of genes located in the CNVs. Further, the subsequently performed correlation analysis (Cohen’s weighted Kappa) revealed a significantly (*p* < 0.05) positive correlation between copy number and gene expression in three of the four brain regions examined (CeA: *K* = 0.260, *p* = 0.0028; BLA: *K* = 0.124, *p* = 0.0815; PVN: *K* = 0.270, *p* = 0.0028; Cg: *K* = 0.230, *p* = 0.0056). That is, more copies of a gene result in higher expression levels.

### Candidate genes of anxiety

After having shown that CNVs are likely to influence gene expression, we identified all protein coding genes in genomic regions revealed to differ in copy number between HAB and LAB mice. The resulting 998 (aCGH), 145 (JaxMDGA), and 1,085 (NGS) genes, respectively, are listed in S8a–S8c Table. In order to improve the reliability of candidate genes, we kept only those genes found by all three methods. The remaining 68 genes (S8d Table) were further compared with protein coding genes within CNVs best associated with anxiety-related behavior in CD-1 mice. Therefore, in a first step, all genes (N = 911) found to overlap with CNVs in 64 CD-1 mice were identified (S8e Table). Next, considering only those CNVs that were best associated with the time animals spent on the open arm of the EPM (nominal *p*-values < 0.1, or < 0.05), the latter list was reduced to 78 genes (S8f Table). Finally, we compared those 78 genes with the 68 genes found inside regions of HAB/LAB CNVs. Among the two sets 15 genes overlapped. Based on a permutation test with 10,000 permutations, the overlap was shown to be significant (*p* = 0.0051). These 15 genes were considered promising candidate genes of anxiety-related behavior and are shown in Table 1.

**Table 1:** Protein coding genes in genomic regions of CNVs detected in HAB/LAB and CD-1 mice.

Gene					CNV (in CD-1 mice)			Association
Symbol	MGI ID	Chr	Start	End	Chr	Start	End	nominal *p*-value
Sirpb1a	2444824	3	15371819	15426504	3	15340258	15819607	0.01213
Sirpb1b	3779828	3	15495754	15575065	3	15340258	15819607	0.01213
Sirpb1c	3807521	3	15695145	15748528	3	15340258	15819607	0.01213
Skint4	2444425	4	111744621	111840681	4	111745396	112286229	0.04514
Skint3	3045331	4	111904850	111973073	4	111745396	112286229	0.04514
Skint9	3045341	4	112058574	112106590	4	111745396	112286229	0.04514
Skint6	3649262	4	112908844	112959568	4	112348832	113968233	0.02412
Skint5	3650151	4	113613249	113672102	4	112348832	113968233	0.02412
Skint11	2685415	4	113835989	113917633	4	112348832	113968233	0.02412
C230055K05Rik	2441896	5	105242188	105288830	5	105034912	105359320	0.03593
Tcp10b	98542	17	13253977	13275092	17	13259210	13301725	0.07376
Gm10512	3642173	17	13397908	13399077	17	13398037	13649185	0.07250
Smok2a	1351487	17	13414054	13420524	17	13398037	13649185	0.07250
Smok2b	3037705	17	13421718	13430055	17	13398037	13649185	0.07250
Gm9880	3711246	17	13547438	13569717	17	13398037	13649185	0.07250

All genes listed overlap both CNVs in HAB/LAB mice detected with aCGH, JaxMDGA and NGS, and CNVs in CD-1 mice which were best associated with the time the animals spent on the open arm of the EPM (nominal *p*-value < 0.1).

Detailed data not shown in this manuscript are available upon request.

## Discussion

In recent years, multiple methods have been developed to screen for CNVs in genome-wide approaches. Nevertheless, CNV detection remains a challenging task since results do not only depend on the method employed but also on the applied algorithm, and no “gold standard” algorithm has been established so far (see, for example, refs. [57, 59–62]). We consider it very important that the reader is aware of the fact that there is neither a generally accepted “gold standard” for a CNV detection method nor for an algorithm, which means that all data generated by just a single method should be regarded and handled with care. This applies not only to our study but also to others. We would like to point out that our association study in CD-1 mice is based on CNV data generated by JaxMDGA solely. Although results were generated thoroughly, they depend on the bias of the applied method and algorithm. Using two probe-based high-density genotyping arrays (aCGH, JaxMDGA) and one whole-genome next-generation sequencing approach (NGS) to reveal CNVs in the HAB/LAB mouse model, we could overcome the limitations of a single approach and thus increase the reliability of the CNV data obtained (S2 Table). The use of multiple processing pipelines and/or data sources is also frequently employed in SNP calling from NGS data in order to improve the reliability of called SNPs and to reduce the chance of false-positive calls [63]. However, the precise breakpoints of CNVs in HAB/LAB mice are likely to be somewhere in between the results of the three applied detection methods.

After having detected the CNVs in HAB and LAB mice, we were further interested in their functional impact. Since effects of CNVs on gene expression levels have been reported before, showing a positive correlation in some cases and a negative one in others [18, 64, 65], we wanted to know, first, if CNVs mediate an effect on gene expression in HAB/LAB mice and, second, if there is a positive or negative correlation between them. Therefore, we compared CNV data with expression data resulting from a genome-wide gene expression analysis. The reliability of expression data was demonstrated by qPCR, with most qPCR outcomes confirming the results of the microarray analysis. Reasons for contradictory findings in array and qPCR data due to a potential method-specific bias are manifold and have already been discussed before by others [66, 67]. We, first, were able to demonstrate that a significant number of genes showing expression differences between HAB and LAB mice were located in CNV regions and, second, found a positive correlation in CeA, Cg and PVN. This outcome is in accordance with a study of Henrichsen and colleagues [56], reporting that the expression of genes within CNVs tends to correlate with changes in copy number. The authors suggested CNVs to “play an even more important role with respect to normal phenotypic variation and risk of complex disease than previously anticipated”. Our study clearly supports this suggestion. Consequently, CNVs should be considered as an influencing factor of gene expression and hence phenotypic variations with respect to anxiety phenotypes. The fact that we could not show a significant correlation between gene dosage and relative expression levels in the BLA might be explained by the CNVs’ modes of action themselves, as reviewed in a recent publication [23]. For example, CNVs might, on one hand, increase gene expression simply by altering gene dosage, and, on the other hand, decrease expression by a negative feedback loop following an increase of the gene products due to the duplication of coding regions [23]. Besides, several environmental and genetic factors such as SNPs, epigenetic factors and others are known to influence gene expression. Thus, especially in case of complex phenotypes, the effects mediated by these factors are likely to interfere with those mediated by CNVs. However, as we could confirm in three of four brain regions tested, the impact of CNVs on gene expression and hence behavioral phenotypes is substantial.

In order to confirm anxiety-relevant CNVs and corresponding genes, we examined CNVs in a second mouse model, the CD-1 outbred mouse model. CNV detection in these mice was performed using an array-based genotyping approach (JaxMDGA). Although multiple computational methods are available to detect CNVs in raw data of genotyping arrays, all commonly applied algorithms compare raw data of each sample against a single reference sample. One consequence of this procedure is an increase in false-negative calls and thus a potential loss of information on CNVs between non-reference samples. We improved CNV calling in the tested 64 CD-1 mice by analyzing the data using each sample once as reference, and thereby conducting all pair-wise comparisons. The mean signal intensities of all JaxMDGA probes within a defined CNV were calculated. In order to keep the number of analysis steps and the related bias as low as possible, we did not define clusters of relative copy numbers, but directly used the mean signal intensities to perform an association analysis of CNVs with anxiety-related behavior. Considering the comparability with HAB/LAB CNV data, the most interesting behavioral parameter is the selection parameter of the HAB/LAB mouse model, that is, the percent time animals spent on the open arm of the EPM. Indeed, we found significant associations of CNVs with this parameter at the nominal *p*-values but not at the threshold corrected for multiple comparisons. This is a well-known phenomenon in genome-wide association studies of complex phenotypes, since common variants with small effect sizes and rare variants are difficult to catch, and multiple-testing correction would often require larger sample sizes than feasible in order to be able to detect these effects [68–70]. Considering the full spectrum of literature available, there should be no doubt that anxiety is induced by complex molecular mechanisms that in turn are influenced by multiple genetic and environmental factors. It was postulated before that the heritability of complex traits is not likely due to some single genes but to multiple genes of small effect size [71]. Thus, specific genes and genetic factors might be less strongly associated with complex traits and diseases than particular patterns of genetic variation and environmental interaction [72]. As a consequence, an association study of a single factor of small effect size with a complex trait leads to significant *p*-values only if other factors are considered in the analysis as well. If such a factor is analyzed solely, significance might get lost, which does not necessarily mean that the single factor *per se* is irrelevant. Almal and Padh [73] have recently postulated that “the implication of CNV on [human] health will have to wait several large-scale correlation studies not only with one CNV but also with permutations and combinations of various likely [genetic and environmental] variations”. To date, however, more complex calculations of associations including all or at least most of the suggested influencing factors are not feasible since the number of these factors pushes the limits of computing capacity. Even though computational methods like those recently developed by Kam-Thong and colleagues [74, 75] facilitate at least the calculation of pair-wise interactions, it will take time until factors of more complex patterns can be offset against each other in a cost- and time-effective manner. Therefore, we decided to use a permutation test to successfully confirm the relevance of genes within CNVs showing nominally significant association, or a statistical trend in their association with anxiety-related behavior.

To our knowledge, the 15 candidate genes revealed in our study were not shown to be linked to anxiety before. However, two of the genes, *Sirpb1a* and *Sirpb1c*, have human equivalents that belong to the so called *SIRP* family. Genes of that large family encode proteins involved in the regulation of signals defining different physiological and pathological processes [76]. *SIRP* family members were suggested to be involved in the activation of the MAPK pathway [77, 78], which was not only shown to play a role in cell differentiation and survival, growth control and cellular adaptation to chemical and physical stress [79–82], but also to be linked to anxiety and depression [83–85]. Thus, further studies on the connection of *Sirpb* genes, the MAPK pathway, and anxiety might be promising.

Interestingly, the *Glo1* gene, a gene within a large and common CNV [18, 64], was not amongst our 15 candidate genes. The *Glo1* gene has been described to influence anxiety-related behavior before [18, 25, 86–89], as discussed in a review by Distler and Palmer [90]. Although we found the CNV including, amongst others, the *Glo1* locus in CD-1 mice and HAB/LAB mice, with more copies and higher expression of *Glo1* in LAB mice, our association study in CD-1 mice did not reveal any influence on anxiety-related behavior. This might be explained by an insufficient statistical power using 64 animals in our study. Using 64 animals only, the power for any QTL analysis is limited, however, the power to show an increase in the respective QTLs based on 64 CD-1 mice and 764 CNVs from an expected number (as done here by a permutation test) is much higher, but maybe not high enough. Since anxiety is a complex trait, the effect of the CNV in this particular experimental setup could be offset by other factors and, thus, many more animals would be required to reveal the effect in an association study. However, similarly, a recent study also could not show an effect of the respective CNV and anxiety-related behavior [91, 92]. Aim of this study was to analyze the influence of *Fkbp5* (FK506 binding protein 5) deficiency on the physiological stress response in *Fkbp5* ^*-/-*^ mice [91]. In a follow-up study analyzing the expression profile of *Fkbp5* ^*-/-*^ and *Fkbp5* ^*+/+*^ mice, an increased expression of *Glo1* mRNA in *Fkbp5* ^*-/-*^ mice was observed, which was shown to result from a co-selection of the *Glo1* duplication with the *Fkbp5* ^*-/-*^allele [92]. Thus, no influence of *Glo1* expression on anxiety-related behavior could be observed in *Fkbp5* ^*-/-*^ and *Fkbp5* ^*+/+*^ mice [91, 92]. Another study using BAC transgenic mice to overexpress *Glo1* demonstrated increased anxiety-related behavior, however, this effect was only observed in mouse lines with the highest copy numbers [88]. In conclusion, Glo1 seems to play a role in the regulation of anxiety-related phenotypes, however its precise effect and the influence of the respective CNV remains to be discovered. As discussed above, complex phenotypes are influenced by a variety of distinct factors; thus, a protein such as Glo1 might be of importance only in the context of a specific genomic background or a certain metabolic state. For example, it was hypothesized that Glo1 affects anxiety-related behavior by controlling levels of methylglyoxal [88, 89]. However, further investigations are required to shed light on the link between Glo1 and anxiety. Although we do not want to extend the discussion at this point, it might also be of interest to examine the relationship between *Glo1* and depression-like behavior in future, since our association study of CD-1 mice showed promising results in the TST (see S5 Table, CNV No. 680).

Taken together, our study provides an extensive catalogue of CNVs and corresponding genes potentially linked to anxiety-related behavior in CD-1 mice. Even though their precise role remains to be investigated, we suggest that these loci might be of interest for future studies focusing on biomarkers of anxiety. Furthermore, with the revelation of CNVs in CD-1 mice, we provide the basis for further investigations of the effects of CNVs in general.

## Supporting Information

S1 Fig Breakpoint definition of CNVs in CD-1 mice. If regions defined as CNVs (orange lines) by applying the „simple CNV”function showed a huge overlap between several sample comparisons, their breakpoints (black dashed lines) were unitized to consider the region as one CNV only. New breakpoints are indicated by green lines. S1 = sample 1, S2 = sample 2, S3 = sample 3.
(TIF)

S2 Fig Q-Q plots of the nominal *p*-values resulting from the associations of CNVs with distinct behavioral parameters. Each behavioral parameter is shown in a distinct plot. Expected *p*-values (x-axis) are plotted against observed *p*-values (y-axis) in logarithmic scale.
(PDF)

S3 Fig Log2 signal intensity ratios of aCGH probes in HAB/LAB mice. The signal ratio of each probe (black dots) refers to the signal intensity of HAB versus LAB sample. Segments defined by “segMNT” are indicated in red. Genomic position on chromosome 17 is shown on the x-axis.
(TIF)

S4 Fig Log2 signal intensity ratios of JaxMDGA probes in HAB/LAB mice. The signal ratio of each probe (black dots) refers to the signal intensity of HAB versus LAB sample. CNVs defined by “simpleCNV” are indicated in red. Genomic position on chromosome 17 is shown on the x-axis.
(TIF)

S5 Fig Fold change of CNVs (NGS) plotted against genomic positions on chromosome 17. CNVs in HAB/LAB mice discovered by “CNVfinder” on Chromosome 17. The x-axis marks the genomic location of the CNV on Chromosome 17. The y-axis corresponds to the log2(fold change). Positive values indicate more copies in HAB than in LAB. Likewise, negative values indicate more copies in LAB compared to HAB.
(TIF)

S6 Fig Genomic positions of CNVs on chromosomes 1–19, X and Y. Chromosomes are indicated by thick grey lines with basepair information shown on the x-axis. Depending on the detection method, CNVs in HAB/LAB mice are depicted in orange (aCGH), dark red (JaxMDGA) and red (NGS), respectively. Data displayed above the grey line represent a copy number gain in HAB vs. LAB animals, data below a copy number loss. Data printed on the grey line show CNVs in 64 CD-1 mice, with those highlighted in color that could be associated with anxiety-related behavior (time on open arm of the EPM) with a nominal *p*-value less than 0.1 (light blue) or less than 0.05 (blue). Start points of CNVs are marked by dots and lines are drawn to the end points.
(PDF)

S1 Table Information on primers used for qPCR. The table is sorted by chromosome. Columns show (left to right): chromosome, gene represented by primer, primer orientation, primer sequence (5’ to 3’), melting temperature and size of the resulting PCR product.
(DOC)

S2 Table CNVs in HAB/LAB mice. Table of all CNVs detected by aCGH, JaxMDGA and NGS in HAB/LAB mice. Data are sorted by genomic position. A CNV detected by one method is shown repeatedly if overlapping with more than one CNV detected by another method. The copy number status (gain/ loss) is shown with respect to HAB animals.
(XLS)

S3 Table Comparison of CNV detection methods. *Upper part*: counts of respective CNVs found in HAB/LAB mice. Numbers in parentheses indicate contradictory findings of distinct methods, that is, a copy number loss found by one method and a gain found by at least one other method. Line “overlap both others”shows the number of CNVs defined by the respective detection method that overlap with any other CNV detected by the other two methods. *Part below*: size of respective CNVs in basepairs (bp).
(DOC)

S4 Table Mean normalized intensities of all CNVs in 64 CD-1 mice and results of behavioral tests. **(a)** In the first line the percentage of time the animals spent on the open arm of the EPM is shown. Below the mean normalized intensities of all CNVs (position information in columns A-E) are shown for the respective animals. **(b)** Original data of performed behavioral tests of all 64 animals are shown, Those data after GRAMMAR transformation are provided in **(c)**.
(XLS)

S5 Table Results of the association analysis of CNVs with behavior in CD-1 mice. For each test parameter of all performed behavioral tests (EPM, FST, OF, TST, SRT), the nominal *p*-values and *p*-values corrected for multiple testing using Holm’s correction method are shown. Further columns show information on CNVs’ chromosomal positions and the number of probes on the array (JaxMDGA) targeting the respective CNV.
(XLS)

S6 Table Results of expression microarray. **(a)** All probes of the expression microarray (12,171) that passed the filtering process are listed, including information about their genomic position. Up to three positions are shown if probes could be mapped to the reference genome multiple times. The *p*-values shown indicate differences in expression between HAB and LAB mice (significant if *p* < 0.05) in the respective brain area (CeA, BLA, Cg, PVN). The relative difference in expression is given as “fold change” (foldCh), with positive values indicating more expression in HAB mice. **(b)** All protein coding genes revealed to be differentially expressed between HAB and LAB mice by expression microarray are listed. The columns contain information about (from left to right): gene symbol, gene number (MGI), chromosome, gene start and end position, the number of microarray probes targeting the gene and showing significant expression differences (*p* < 0.05), and, finally, the number of probes showing more and less expression in HAB mice in the respective brain region (CeA, BLA, Cg and PVN).
(XLS)

S7 Table Expression differences of genes tested in qPCR. The first part shows the relative expression rate with standard error (SEM) and *p*-value (calculated by Mann-Whitney-U test) for CeA and BLA, the second part for PVN and Cg. A *p*-value < 0.05 (bold letters) indicates a significant difference in gene expression between HAB and LAB mice; a *p*-value < 0.1 (bold and italic letters) indicates a trend.
(DOC)

S8 Table Protein coding genes in genomic regions of CNVs detected in HAB/LAB (a - d) and CD-1 mice (e,f). Genes in regions of CNVs detected in HAB/LAB mice by **(a)** aCGH, **(b)** JaxMDGA and **(c)** NGS, respectively, are listed and the position information of genes and correspondent CNVs are shown. Genes are listed multiple times if overlapping with more than one CNV. In **(d)** those genes overlapping CNVs detected in HAB/LAB mice by all of the three detection methods are displayed. **(e)** Genes in genomic regions of CNVs detected in 64 CD-1 mice by JaxMDGA are listed and the position information of genes and correspondent CNVs are shown. **(f)** Reduction of table (e) to those genes overlapping CNVs that could be best associated with the time the animals spent on the open arm of the EPM (nominal *p*-values < 0.1, or < 0.05).
(XLS)

S1 Text Supporting Material and Methods. (DOC)
