# Supplementary figures and images for: Connecting Anxiety and Genomic Copy Number Variation: A Genome-Wide Analysis in CD-1 Mice
Source: PLoS One. 2015 May 26;10(5):e0128465. doi: 10.1371/journal.pone.0128465 (PMC4444327; doi:10.1371/journal.pone.0128465)

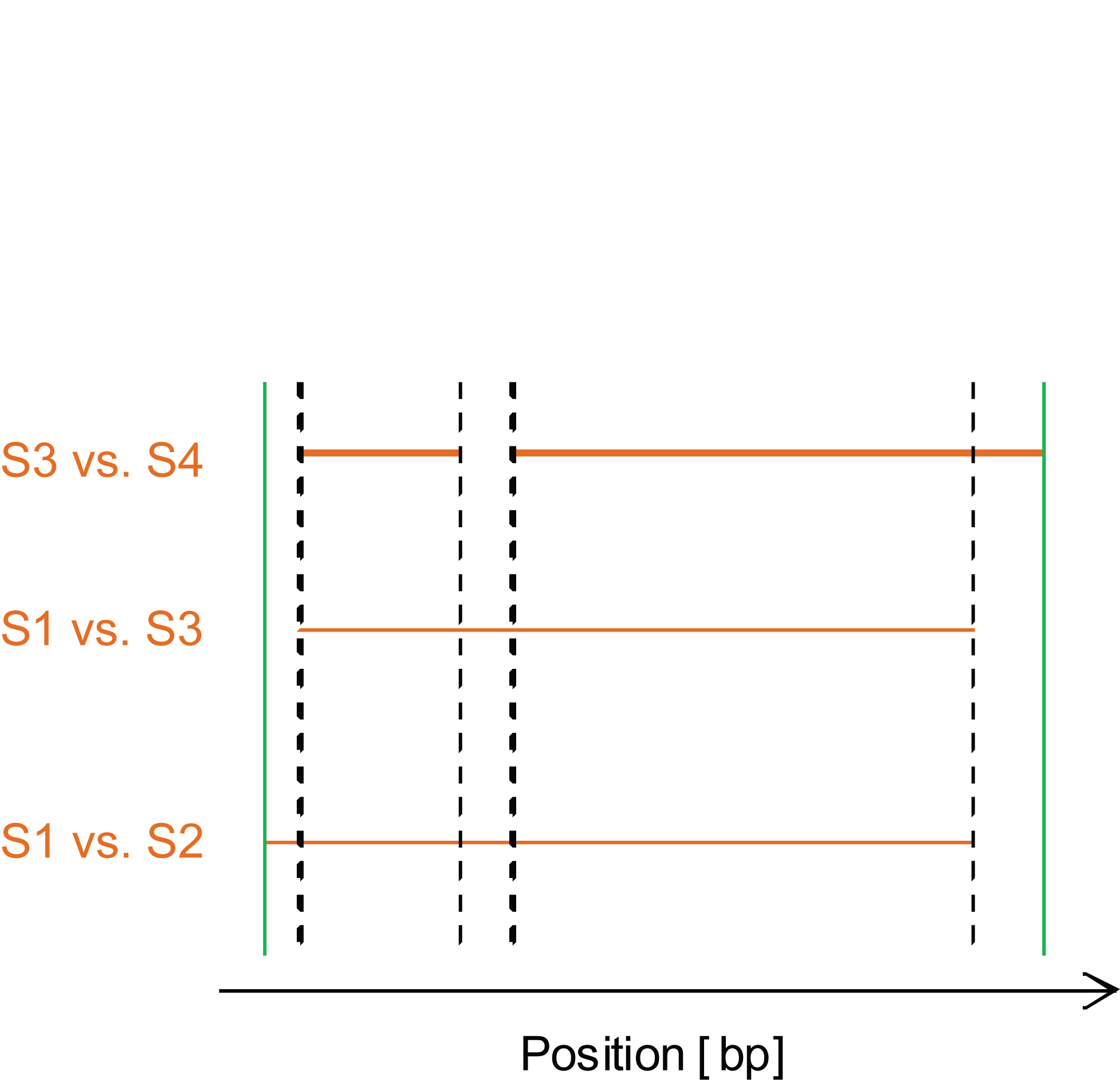

Supplement: S1 Fig — If regions defined as CNVs (orange lines) by applying the „simple CNV”function showed a huge overlap between several sample comparisons, their breakpoints (black dashed lines) were unitized to consider the region as one CNV only. New breakpoints are indicated by green lines. S1 = sample 1, S2 = sample 2, S3 = sample 3. (TIF) [file pone.0128465.s001.tif]

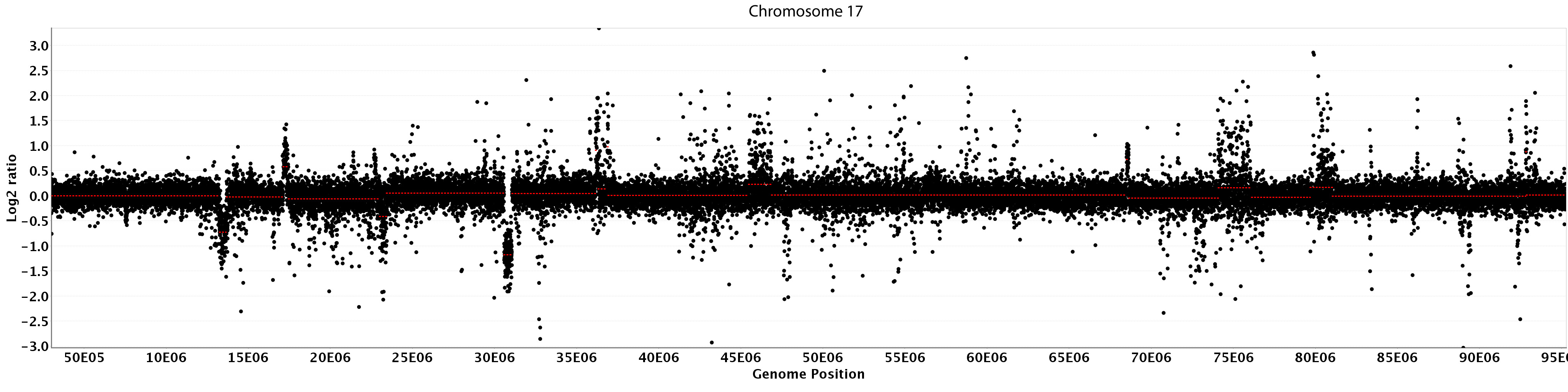

Supplement: S3 Fig — The signal ratio of each probe (black dots) refers to the signal intensity of HAB versus LAB sample. Segments defined by “segMNT” are indicated in red. Genomic position on chromosome 17 is shown on the x-axis. (TIF) [file pone.0128465.s003.tif]

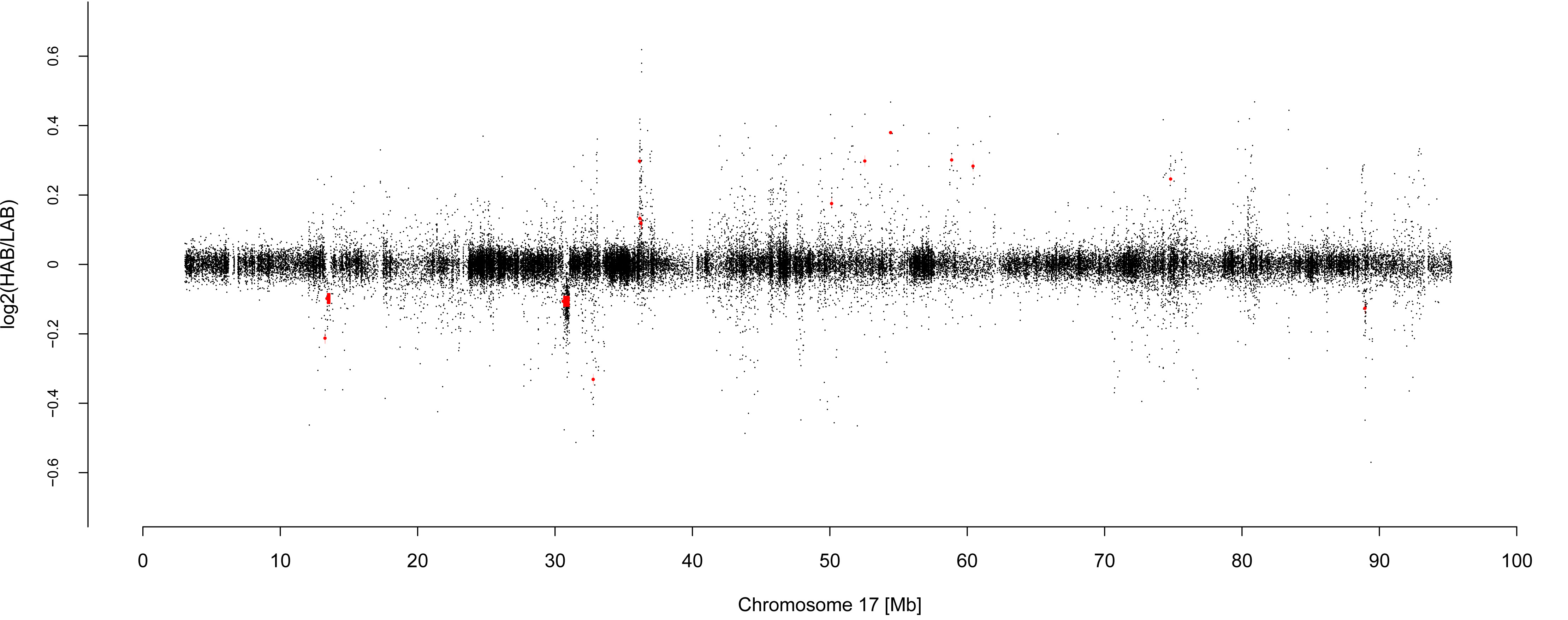

Supplement: S4 Fig — The signal ratio of each probe (black dots) refers to the signal intensity of HAB versus LAB sample. CNVs defined by “simpleCNV” are indicated in red. Genomic position on chromosome 17 is shown on the x-axis. (TIF) [file pone.0128465.s004.tif]

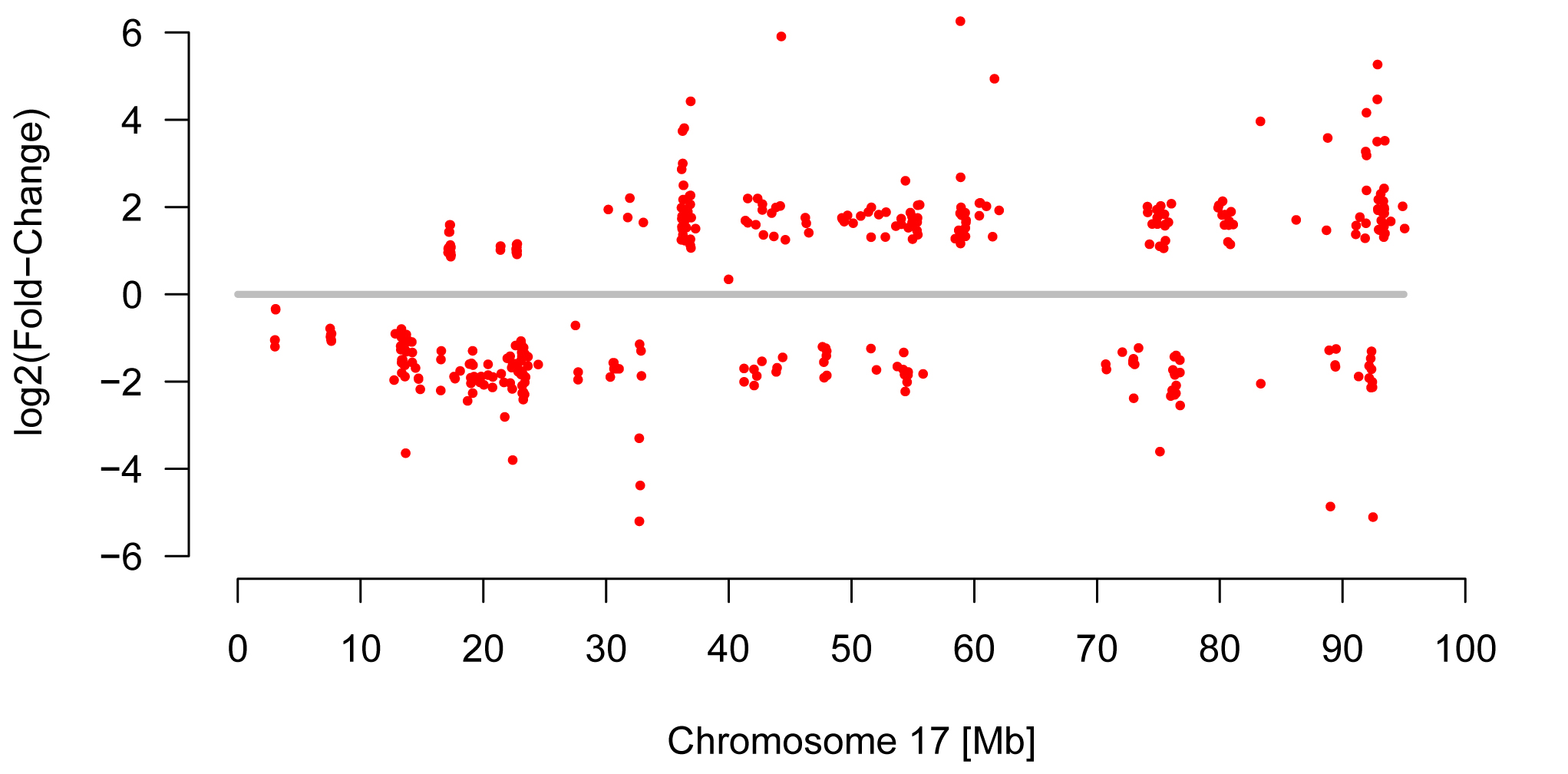

Supplement: S5 Fig — CNVs in HAB/LAB mice discovered by “CNVfinder” on Chromosome 17. The x-axis marks the genomic location of the CNV on Chromosome 17. The y-axis corresponds to the log2(fold change). Positive values indicate more copies in HAB than in LAB. Likewise, negative values indicate more copies in LAB compared to HAB. (TIF) [file pone.0128465.s005.tif]
