# Supplementary material for: Connecting Anxiety and Genomic Copy Number Variation: A Genome-Wide Analysis in CD-1 Mice
Source: PLoS One. 2015 May 26;10(5):e0128465. doi: 10.1371/journal.pone.0128465 (PMC4444327; doi:10.1371/journal.pone.0128465)
Supplement: S2 Fig — Each behavioral parameter is shown in a distinct plot. Expected p-values (x-axis) are plotted against observed p-values (y-axis) in logarithmic scale. (PDF) [file pone.0128465.s002.pdf]

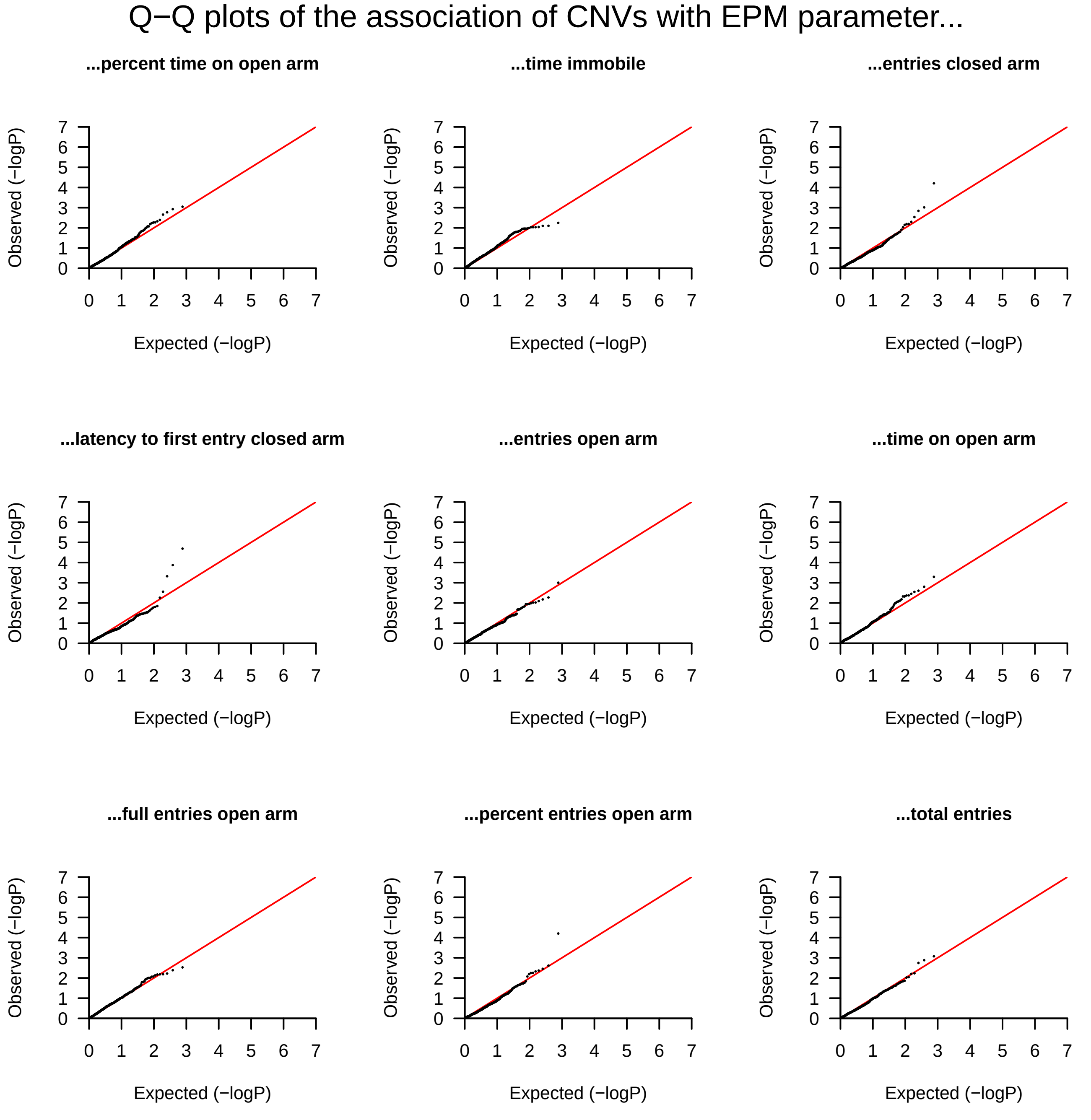
Q−Q plots of the association of CNVs with EPM parameter...
...percent time on open arm
...time immobile
...entries closed arm
...latency to first entry closed arm
...entries open arm
...time on open arm
...full entries open arm
...percent entries open arm
...total entries
Observed (−logP)
Expected (−logP)

Q−Q plots of the association of CNVs with transf. OF parameter...

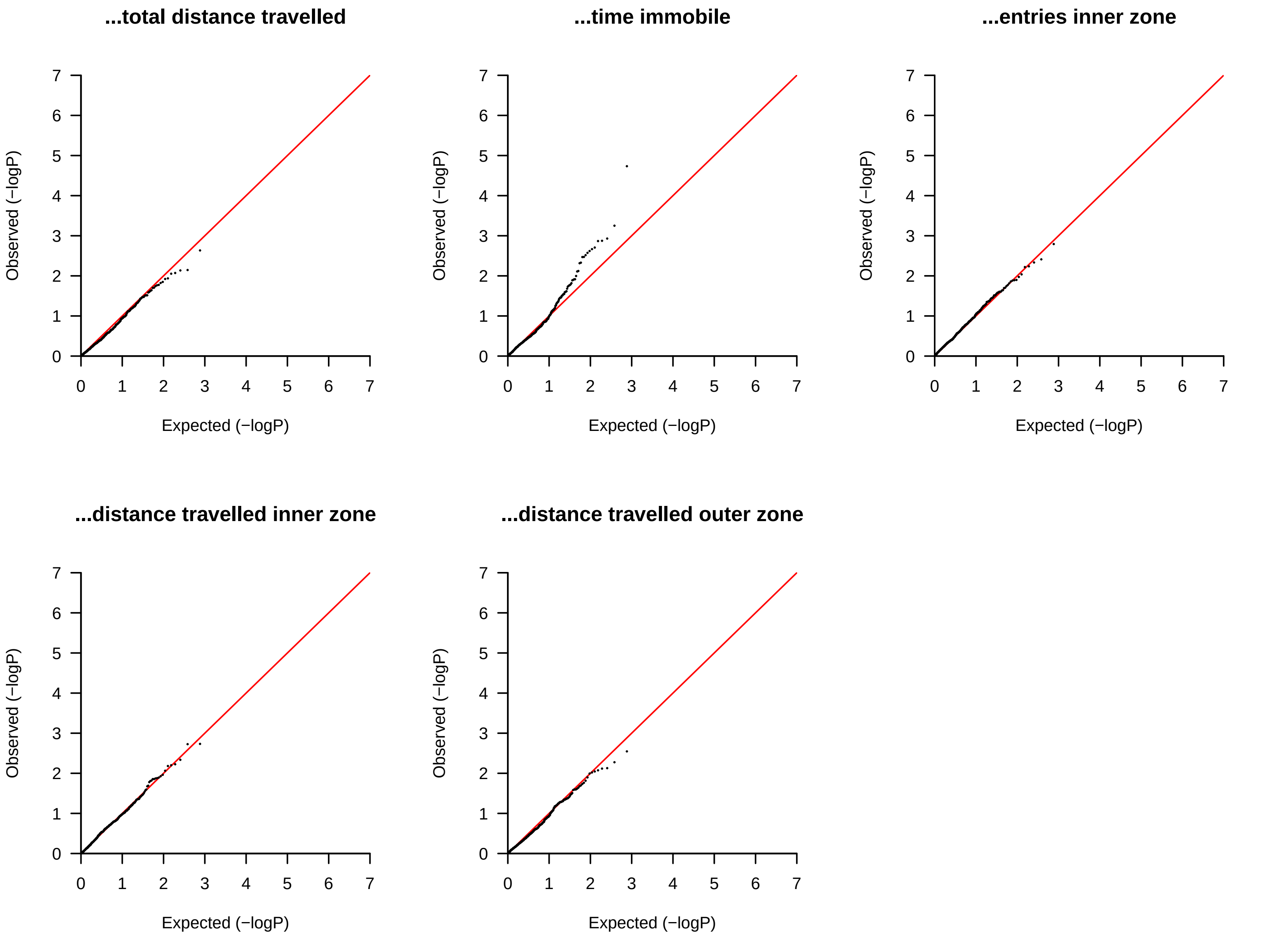

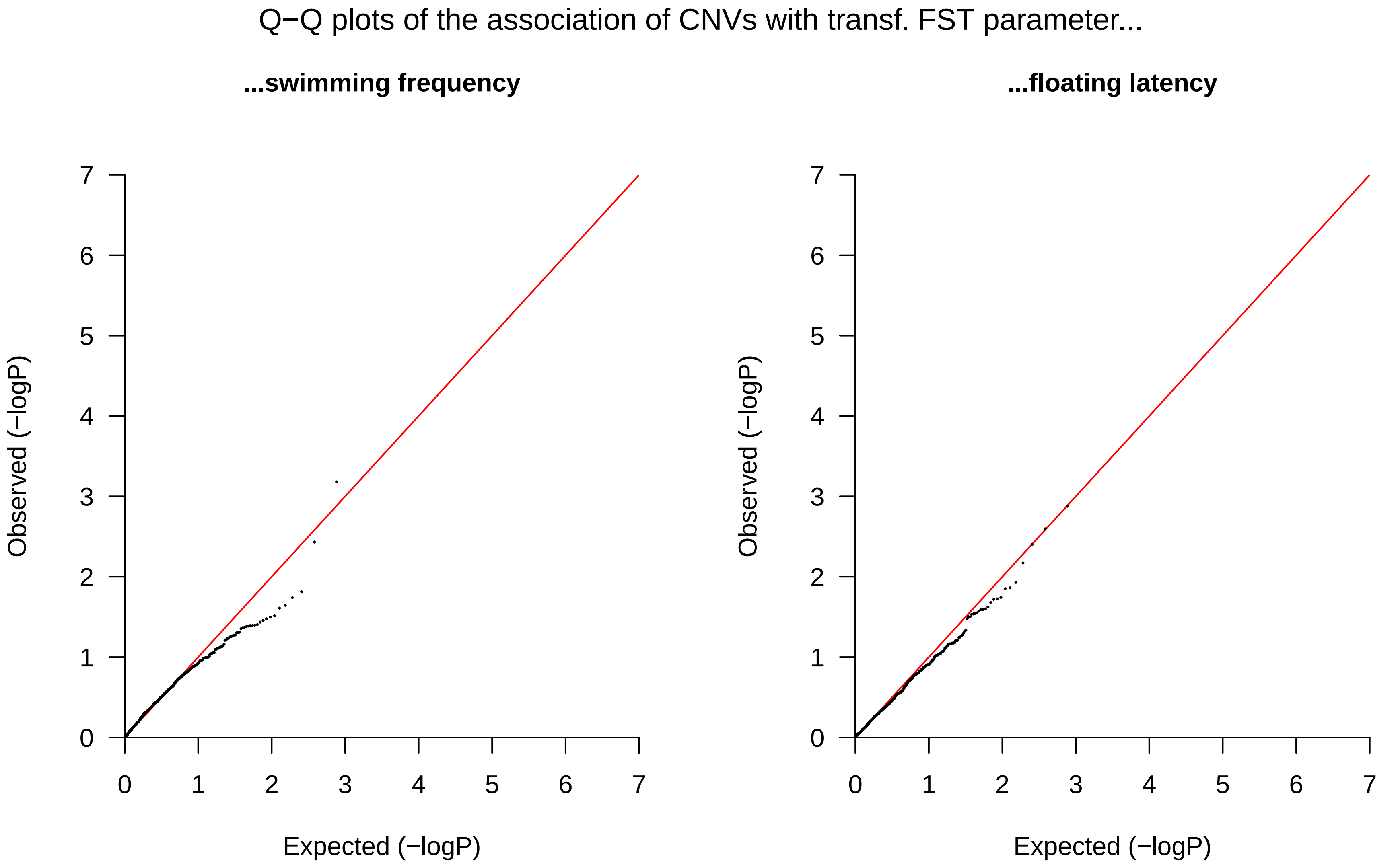
Q−Q plots of the association of CNVs with transf. FST parameter...
...swimming frequency
...floating latency
Observed (−logP)
Expected (−logP)
0
1
2
3
4
5
6
7

Q−Q plots of the association of CNVs with transf. SRT parameter...

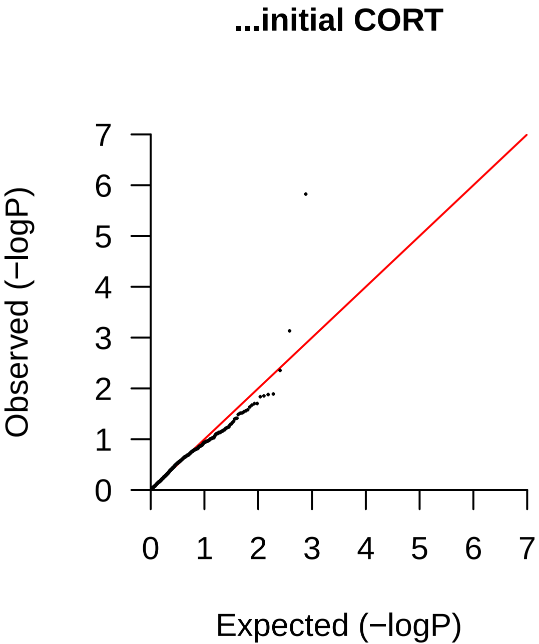

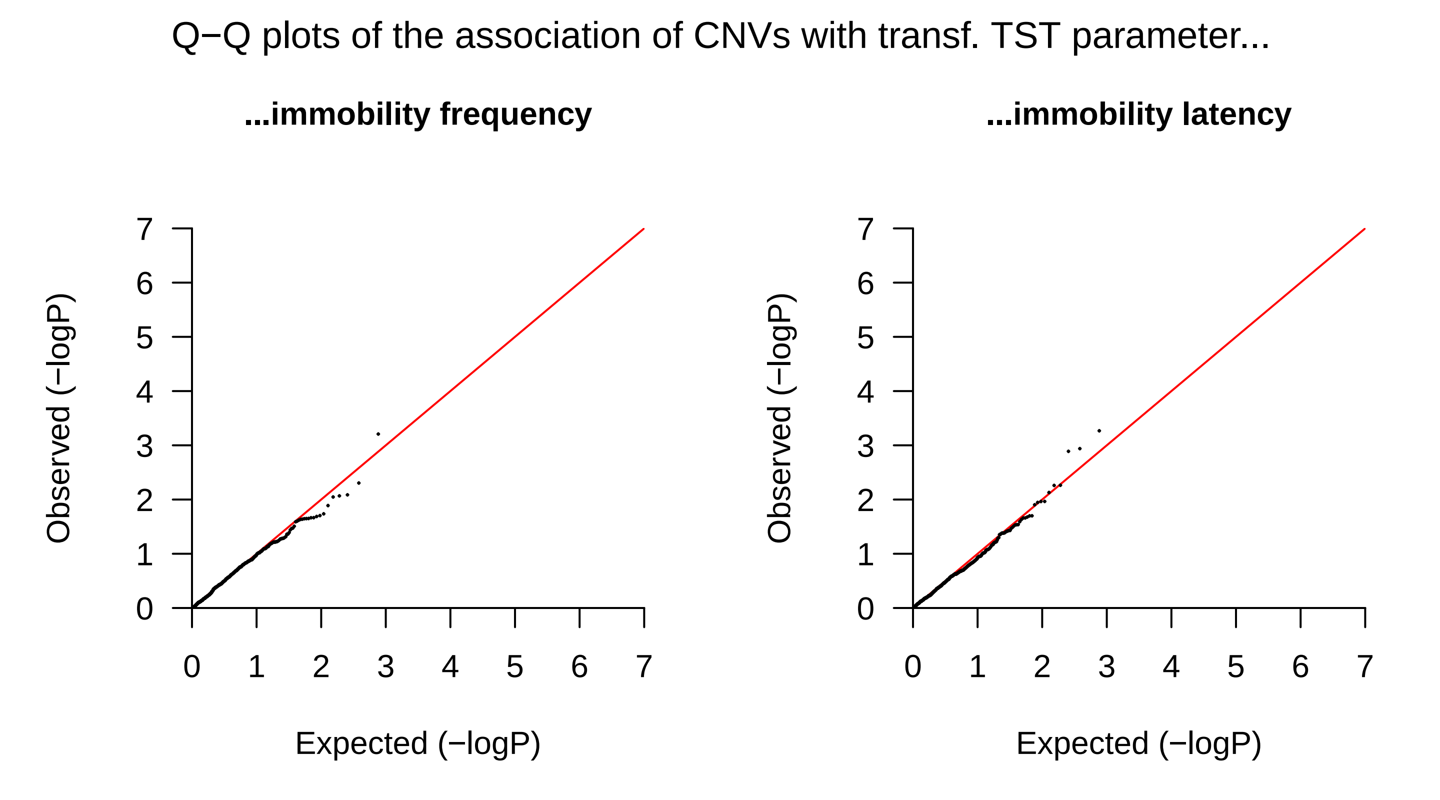

Q−Q plots of the association of CNVs with transf. TST parameter...
...immobility frequency
...immobility latency
Observed (−logP)
Expected (−logP)
0
1
2
3
4
5
6
7
