# Supplementary material for: Connecting Anxiety and Genomic Copy Number Variation: A Genome-Wide Analysis in CD-1 Mice
Source: PLoS One. 2015 May 26;10(5):e0128465. doi: 10.1371/journal.pone.0128465 (PMC4444327; doi:10.1371/journal.pone.0128465)
Supplement: S6 Fig — Chromosomes are indicated by thick grey lines with basepair information shown on the x-axis. Depending on the detection method, CNVs in HAB/LAB mice are depicted in orange (aCGH), dark red (JaxMDGA) and red (NGS), respectively. Data displayed above the grey line represent a copy number gain in HAB vs. LAB animals, data below a copy number loss. Data printed on the grey line show CNVs in 64 CD-1 mice, with those highlighted in color that could be associated with anxiety-related behavior (time on open arm of the EPM) with a nominal p-value less than 0.1 (light blue) or less than 0.05 (blue). Start points of CNVs are marked by dots and lines are drawn to the end points. (PDF) [file pone.0128465.s006.pdf]

A)

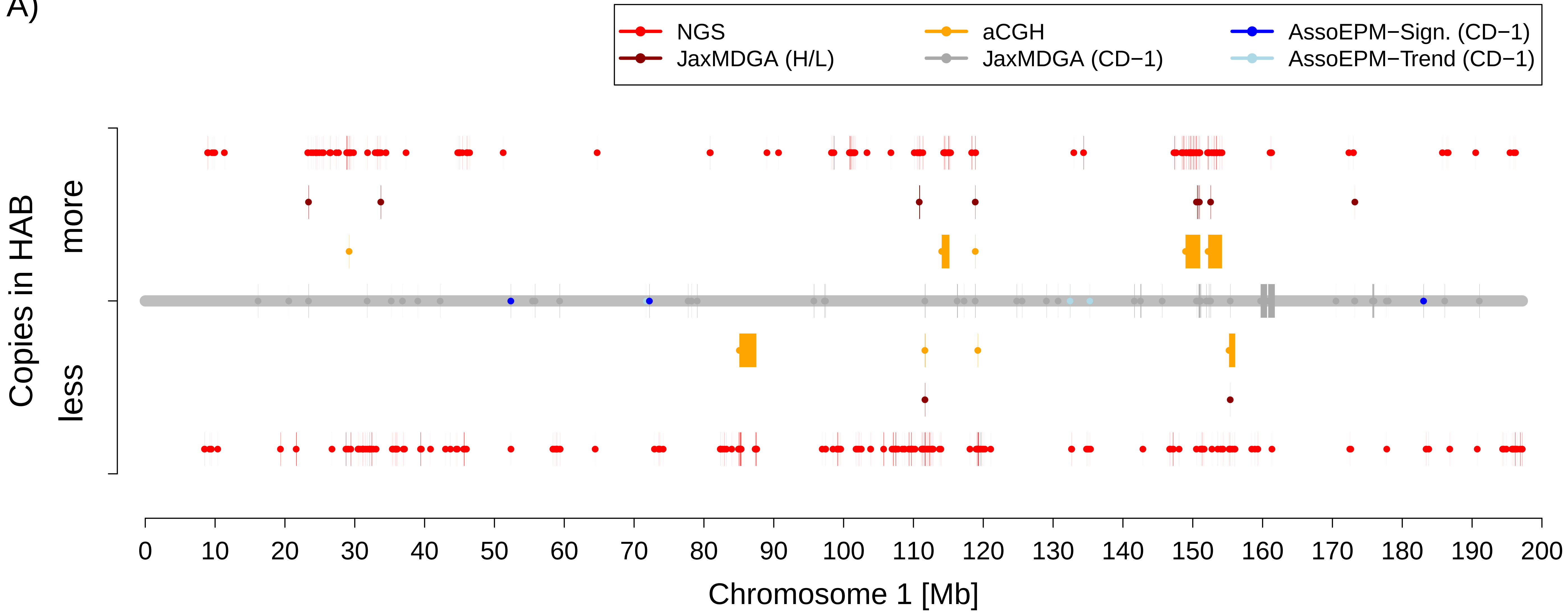
NGS
JaxMDGA (H/L)
aCGH
JaxMDGA (CD−1)
AssoEPM−Sign. (CD−1)
AssoEPM−Trend (CD−1)
Copies in HAB
more
less
0
10
20
30
40
50
60
70
80
90
100
110
120
130
140
150
160
170
180
190
200
Chromosome 1 [Mb]

B)

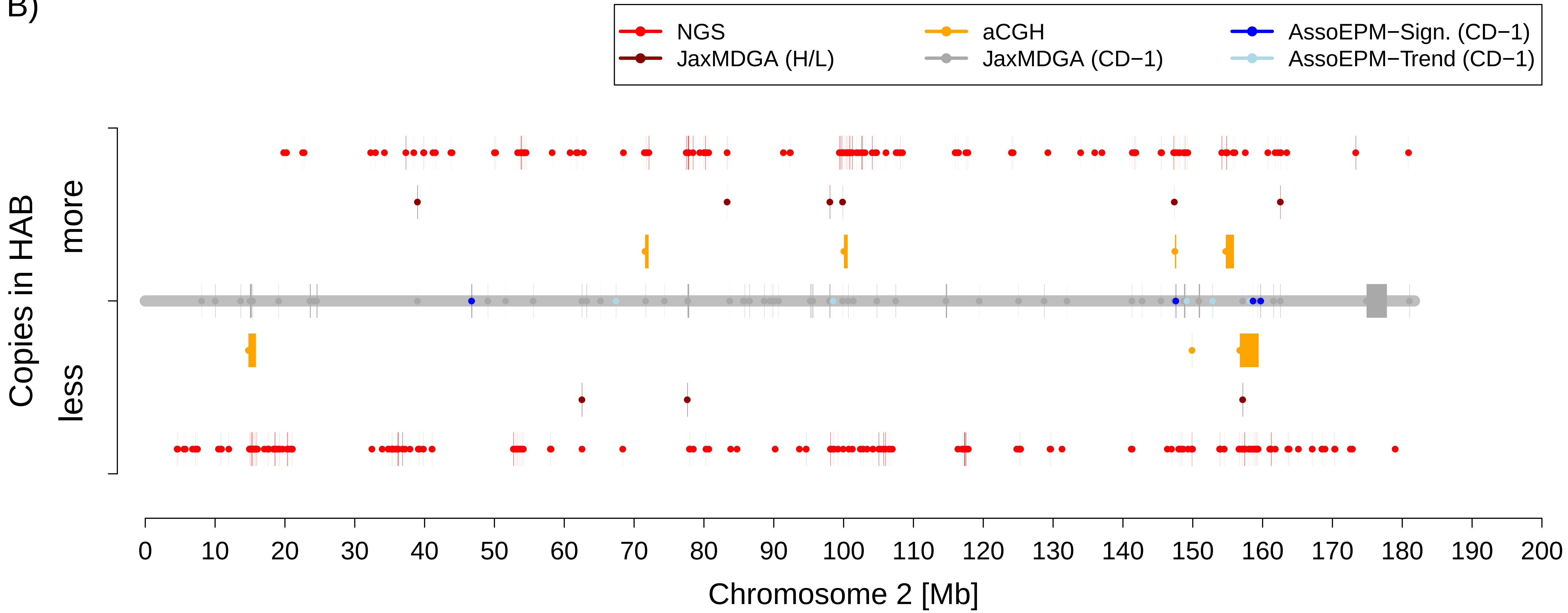
NGS
JaxMDGA (H/L)
aCGH
JaxMDGA (CD−1)
AssoEPM−Sign. (CD−1)
AssoEPM−Trend (CD−1)
Copies in HAB
more
less
0
10
20
30
40
50
60
70
80
90
100
110
120
130
140
150
160
170
180
190
200
Chromosome 2 [Mb]

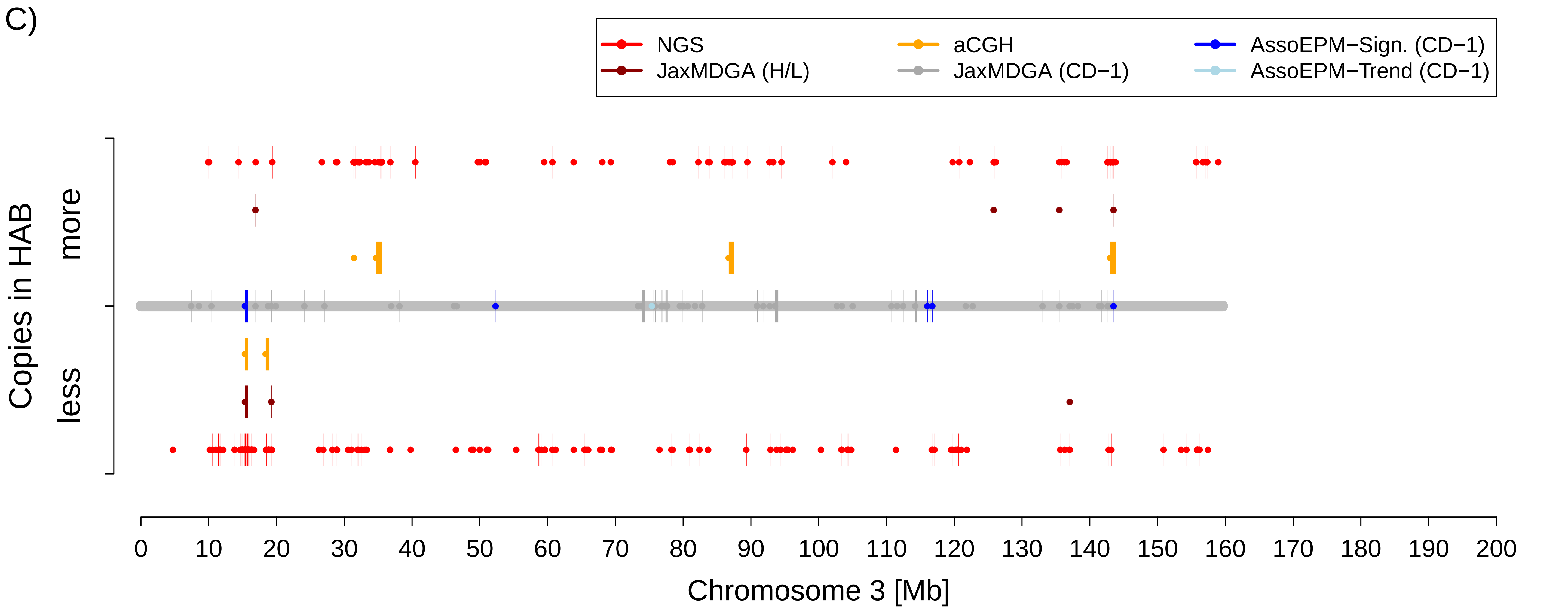
C)
NGS
JaxMDGA (H/L)
aCGH
JaxMDGA (CD−1)
AssoEPM−Sign. (CD−1)
AssoEPM−Trend (CD−1)
Copies in HAB
more
less
0
10
20
30
40
50
60
70
80
90
100
110
120
130
140
150
160
170
180
190
200
Chromosome 3 [Mb]

D)

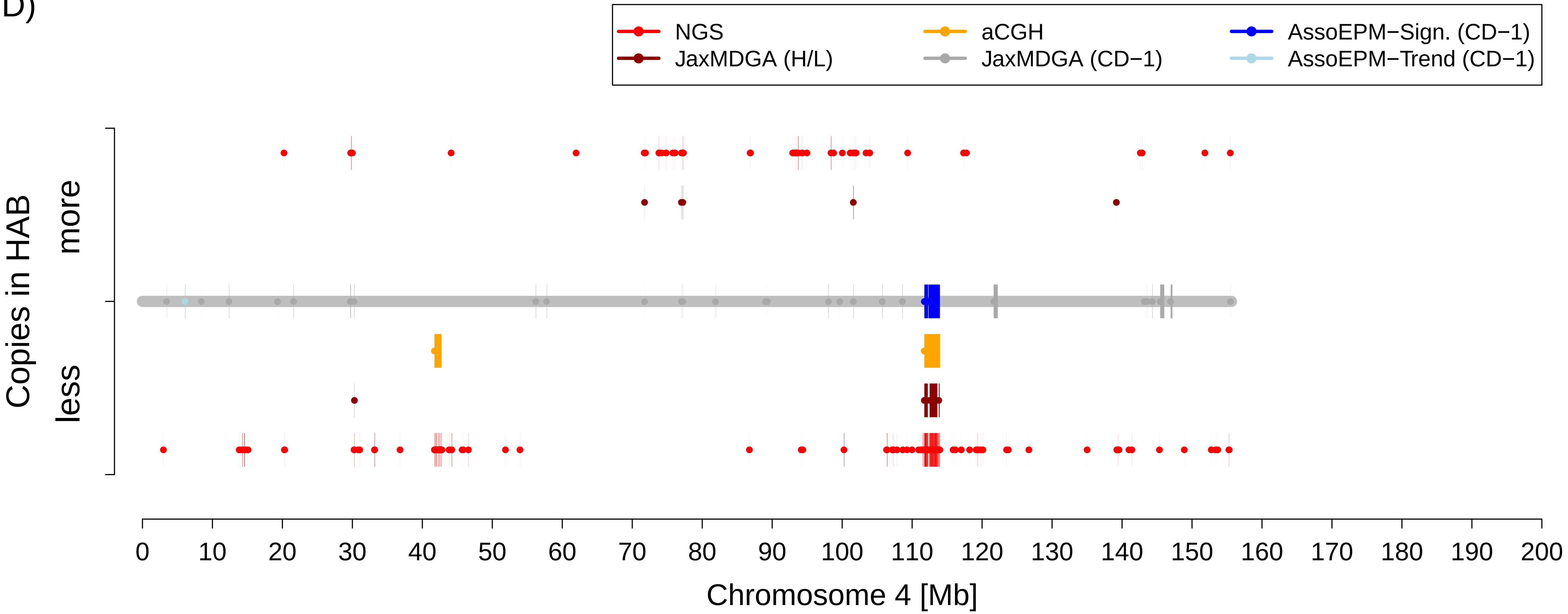
NGS
JaxMDGA (H/L)
aCGH
JaxMDGA (CD−1)
AssoEPM−Sign. (CD−1)
AssoEPM−Trend (CD−1)
Copies in HAB
more
less
0
10
20
30
40
50
60
70
80
90
100
110
120
130
140
150
160
170
180
190
200
Chromosome 4 [Mb]

E)

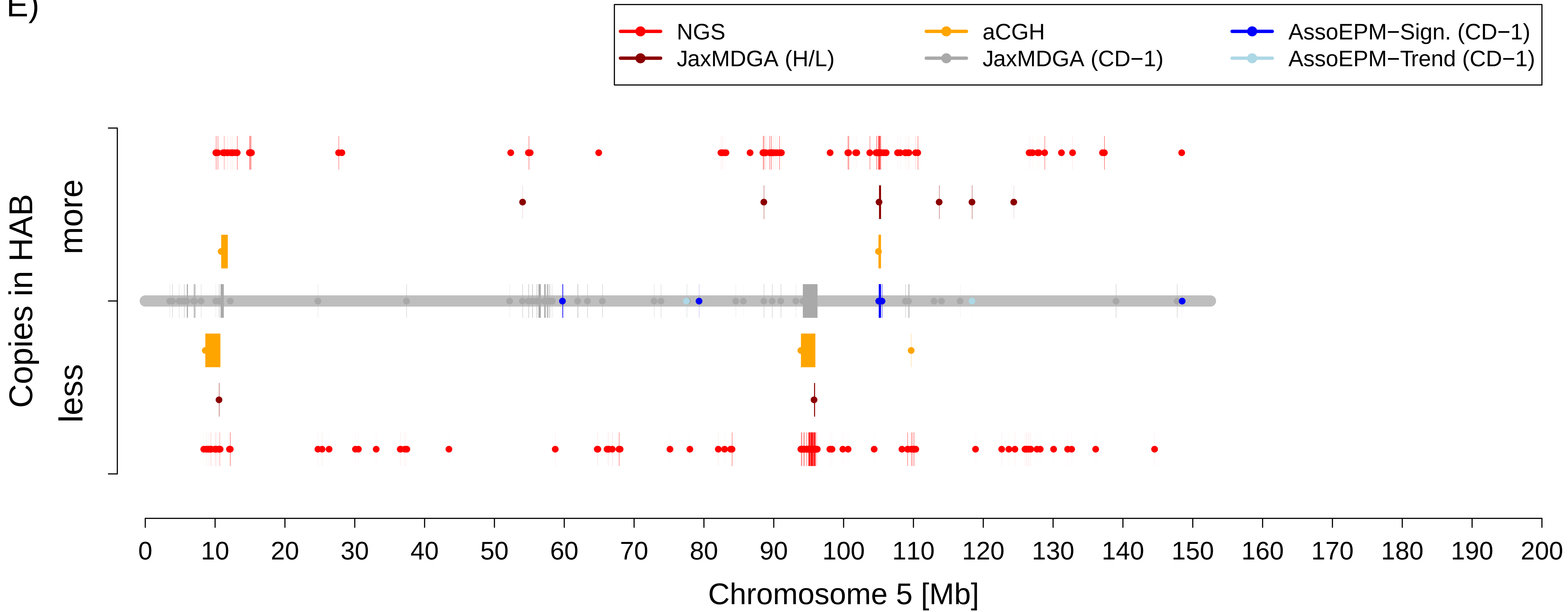

NGS
JaxMDGA (H/L)
aCGH
JaxMDGA (CD−1)
AssoEPM−Sign. (CD−1)
AssoEPM−Trend (CD−1)
Copies in HAB
more
less
0 10 20 30 40 50 60 70 80 90 100 110 120 130 140 150 160 170 180 190 200
Chromosome 5 [Mb]

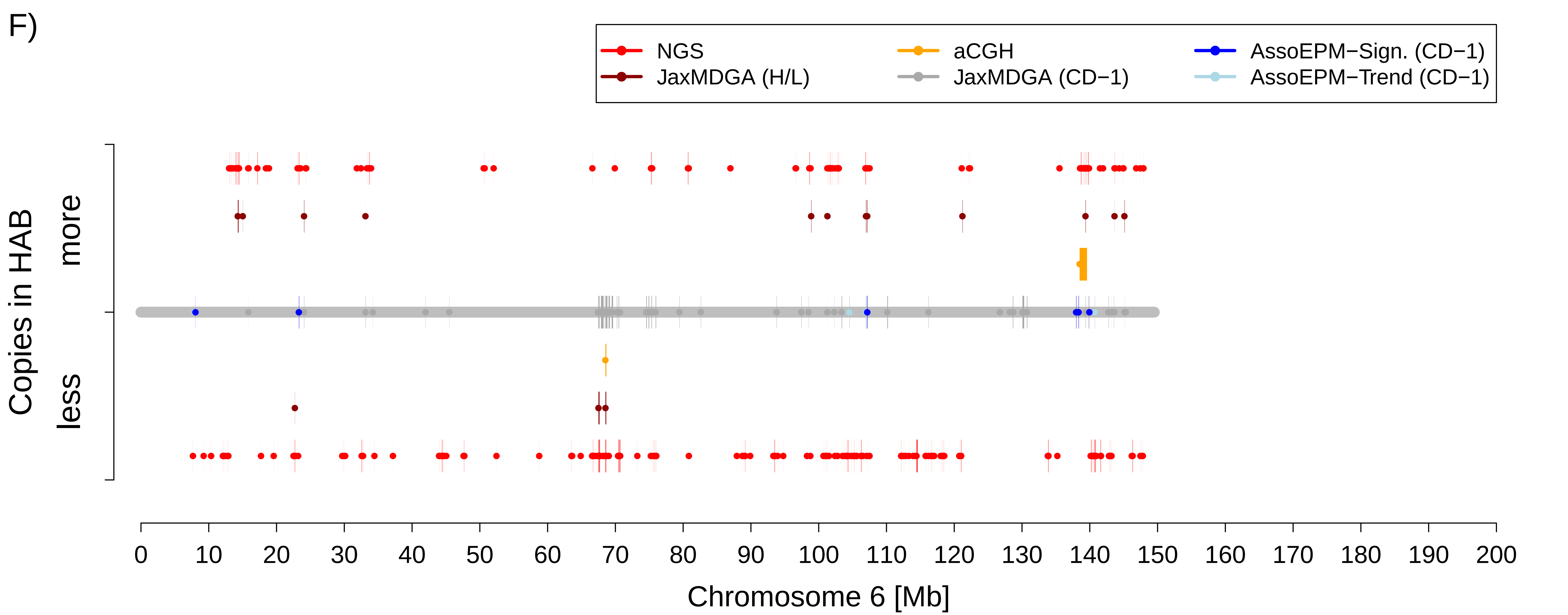
F)
NGS
JaxMDGA (H/L)
aCGH
JaxMDGA (CD−1)
AssoEPM−Sign. (CD−1)
AssoEPM−Trend (CD−1)
Copies in HAB
more
less
0
10
20
30
40
50
60
70
80
90
100
110
120
130
140
150
160
170
180
190
200
Chromosome 6 [Mb]

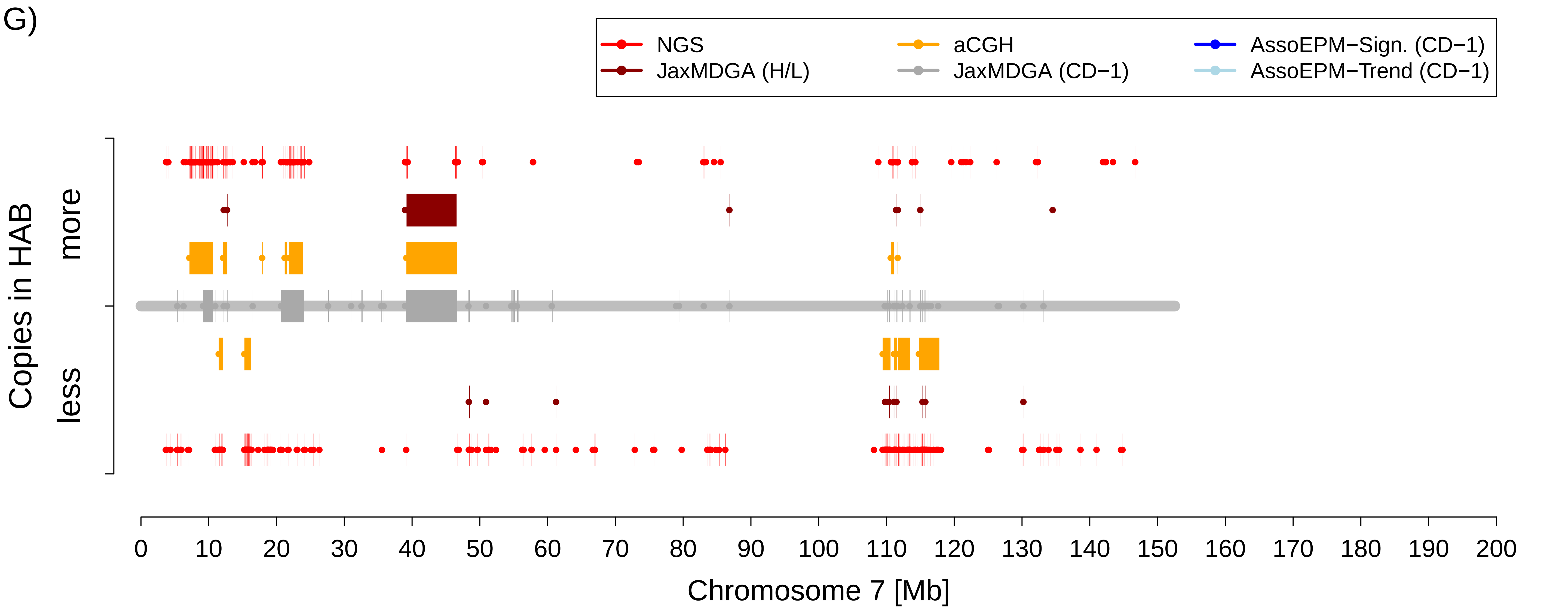

G)
NGS
JaxMDGA (H/L)
aCGH
JaxMDGA (CD−1)
AssoEPM−Sign. (CD−1)
AssoEPM−Trend (CD−1)
Copies in HAB
more
less
0
10
20
30
40
50
60
70
80
90
100
110
120
130
140
150
160
170
180
190
200
Chromosome 7 [Mb]

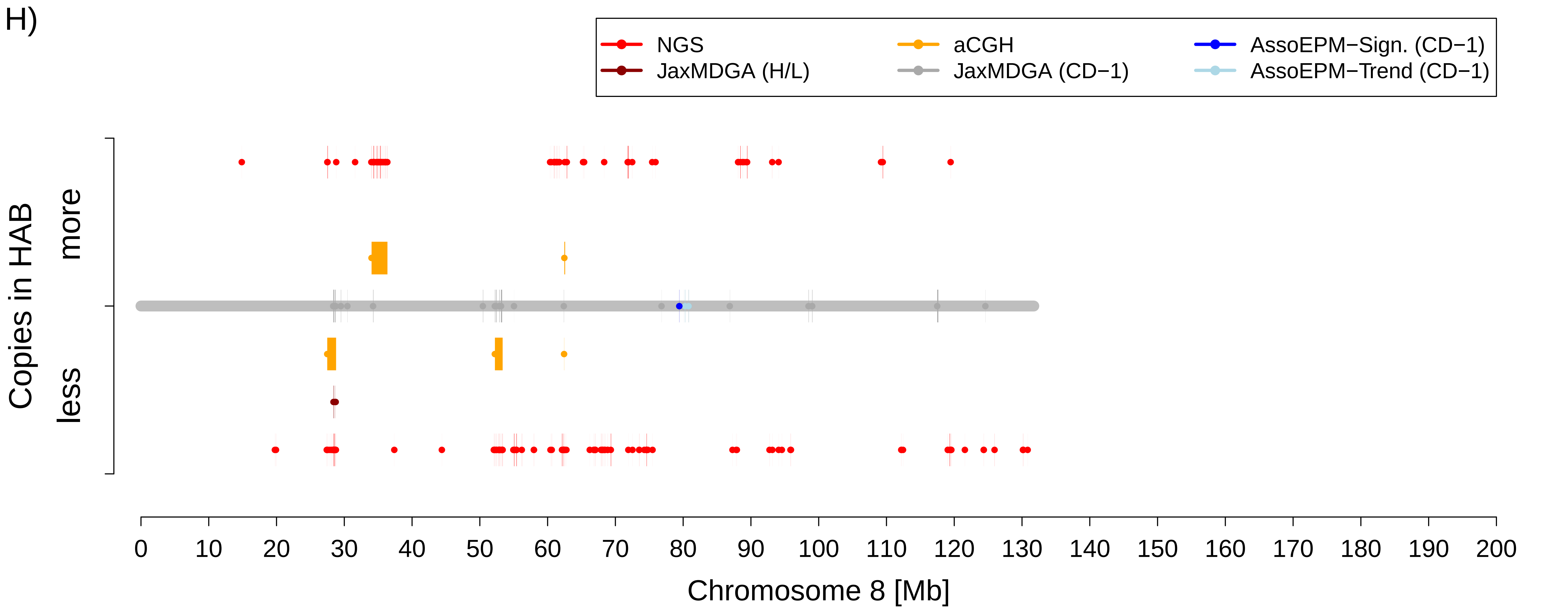
H)
NGS
JaxMDGA (H/L)
aCGH
JaxMDGA (CD−1)
AssoEPM−Sign. (CD−1)
AssoEPM−Trend (CD−1)
Copies in HAB
more
less
0 10 20 30 40 50 60 70 80 90 100 110 120 130 140 150 160 170 180 190 200
Chromosome 8 [Mb]

I)

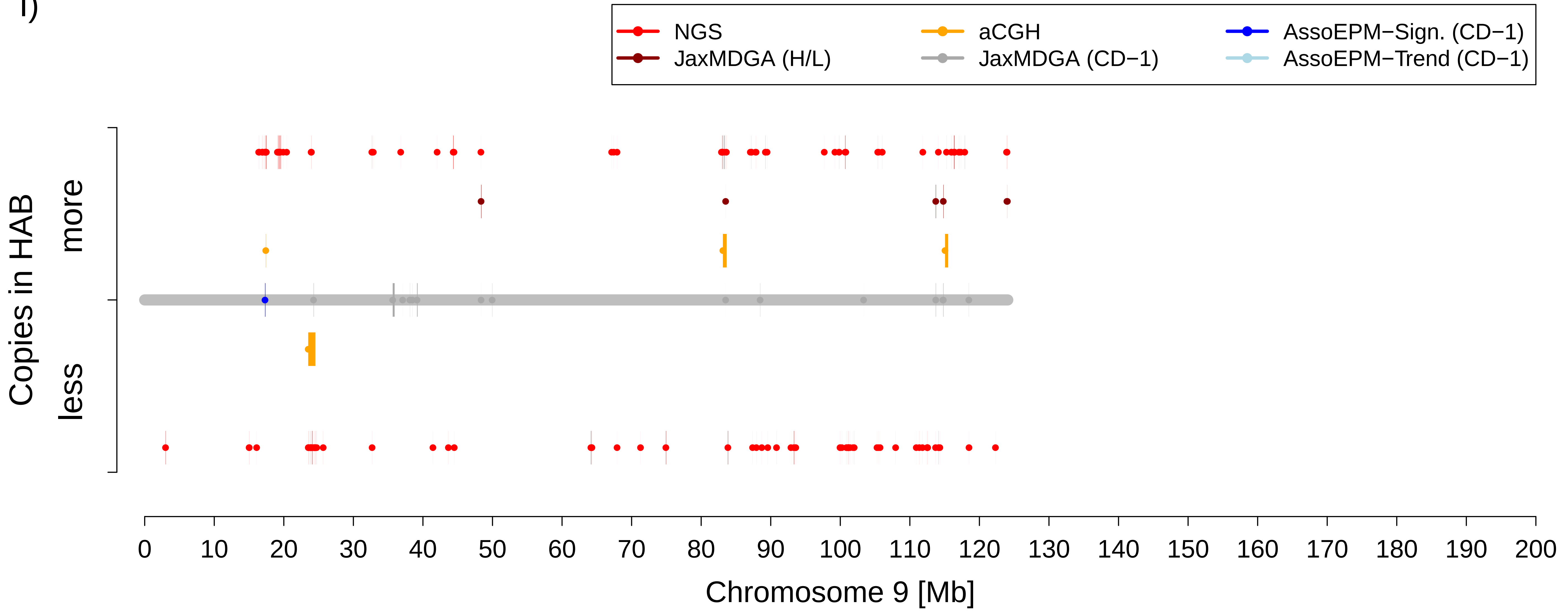

NGS
JaxMDGA (H/L)
aCGH
JaxMDGA (CD−1)
AssoEPM−Sign. (CD−1)
AssoEPM−Trend (CD−1)
Copies in HAB
more
less
0 10 20 30 40 50 60 70 80 90 100 110 120 130 140 150 160 170 180 190 200
Chromosome 9 [Mb]

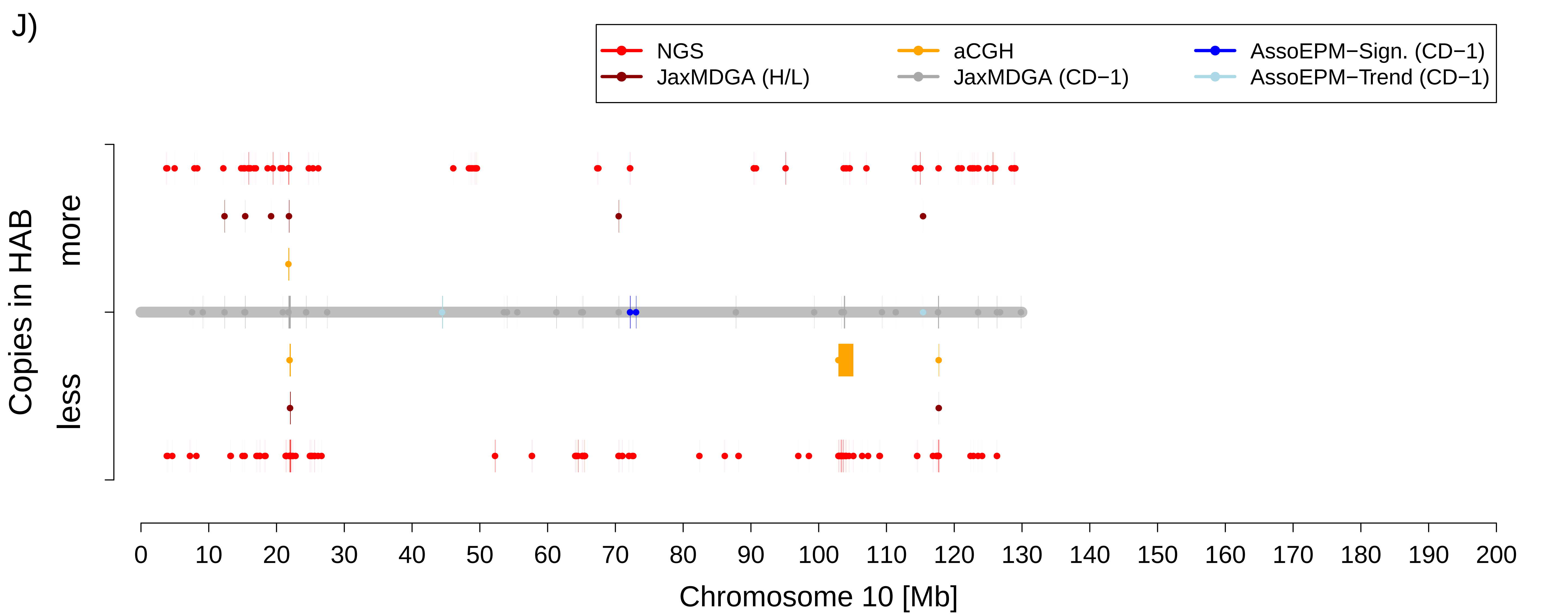
J)
NGS
JaxMDGA (H/L)
aCGH
JaxMDGA (CD−1)
AssoEPM−Sign. (CD−1)
AssoEPM−Trend (CD−1)
Copies in HAB
more
less
0
10
20
30
40
50
60
70
80
90
100
110
120
130
140
150
160
170
180
190
200
Chromosome 10 [Mb]

K)

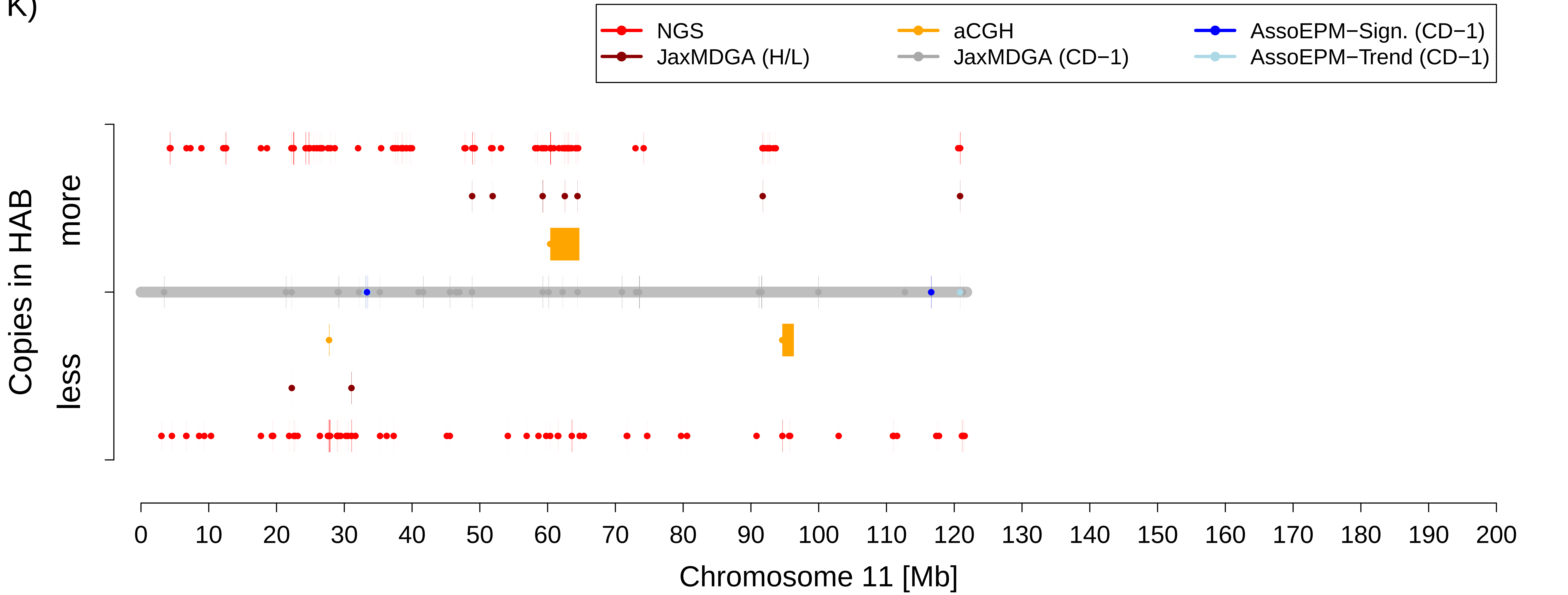
NGS
JaxMDGA (H/L)
aCGH
JaxMDGA (CD−1)
AssoEPM−Sign. (CD−1)
AssoEPM−Trend (CD−1)
Copies in HAB
more
less
0
10
20
30
40
50
60
70
80
90
100
110
120
130
140
150
160
170
180
190
200
Chromosome 11 [Mb]

L)

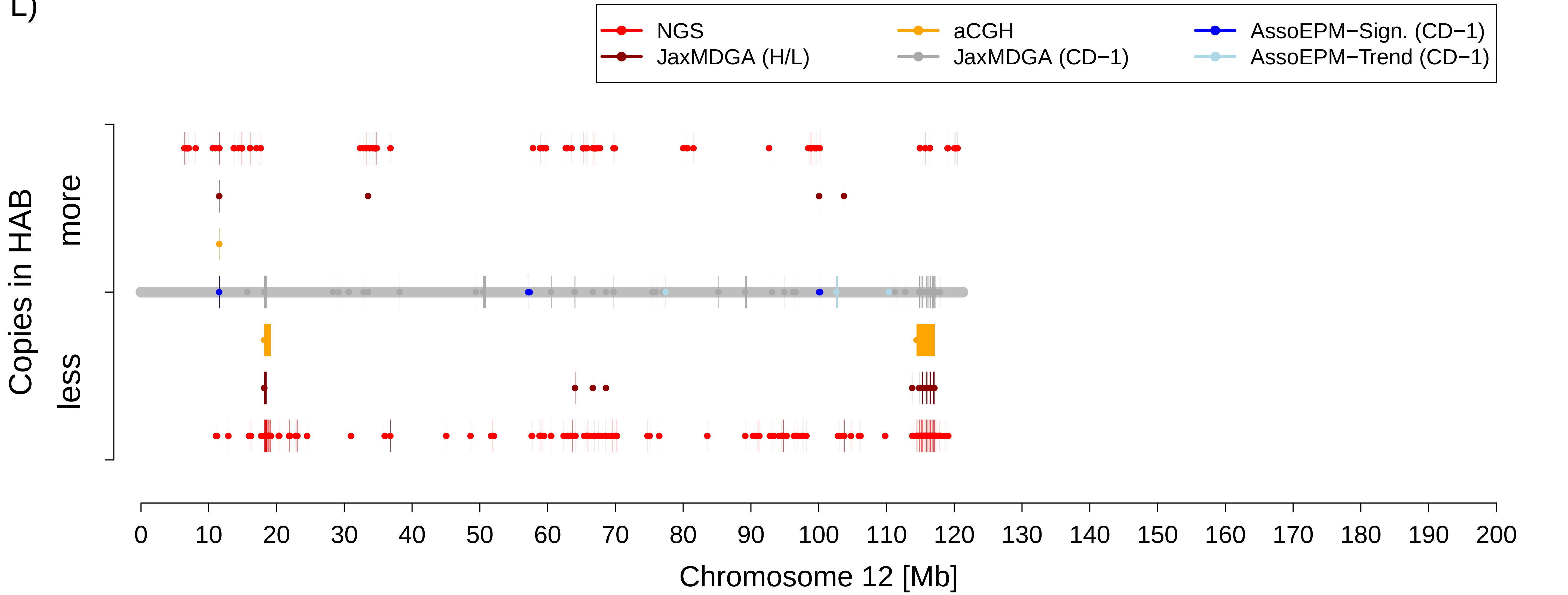
NGS
JaxMDGA (H/L)
aCGH
JaxMDGA (CD−1)
AssoEPM−Sign. (CD−1)
AssoEPM−Trend (CD−1)
Copies in HAB
more
less
0
10
20
30
40
50
60
70
80
90
100
110
120
130
140
150
160
170
180
190
200
Chromosome 12 [Mb]

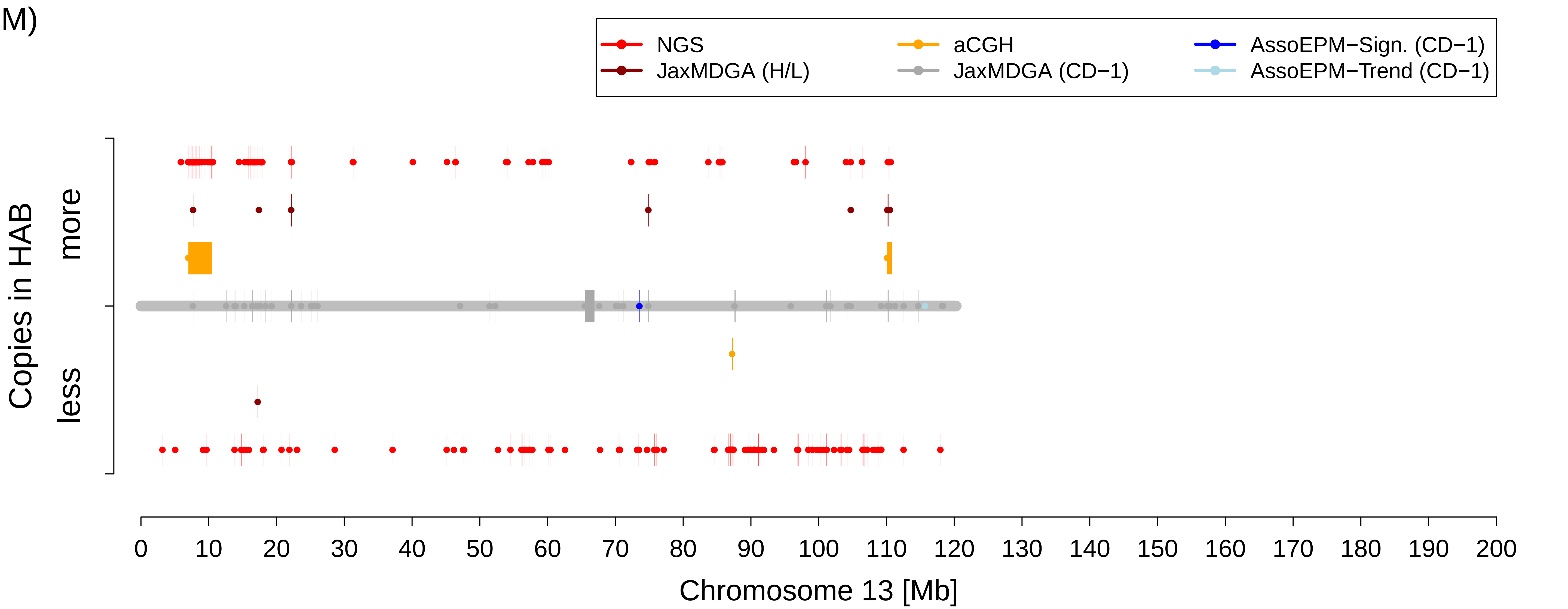
M)
NGS
JaxMDGA (H/L)
aCGH
JaxMDGA (CD−1)
AssoEPM−Sign. (CD−1)
AssoEPM−Trend (CD−1)
Copies in HAB
more
less
0
10
20
30
40
50
60
70
80
90
100
110
120
130
140
150
160
170
180
190
200
Chromosome 13 [Mb]

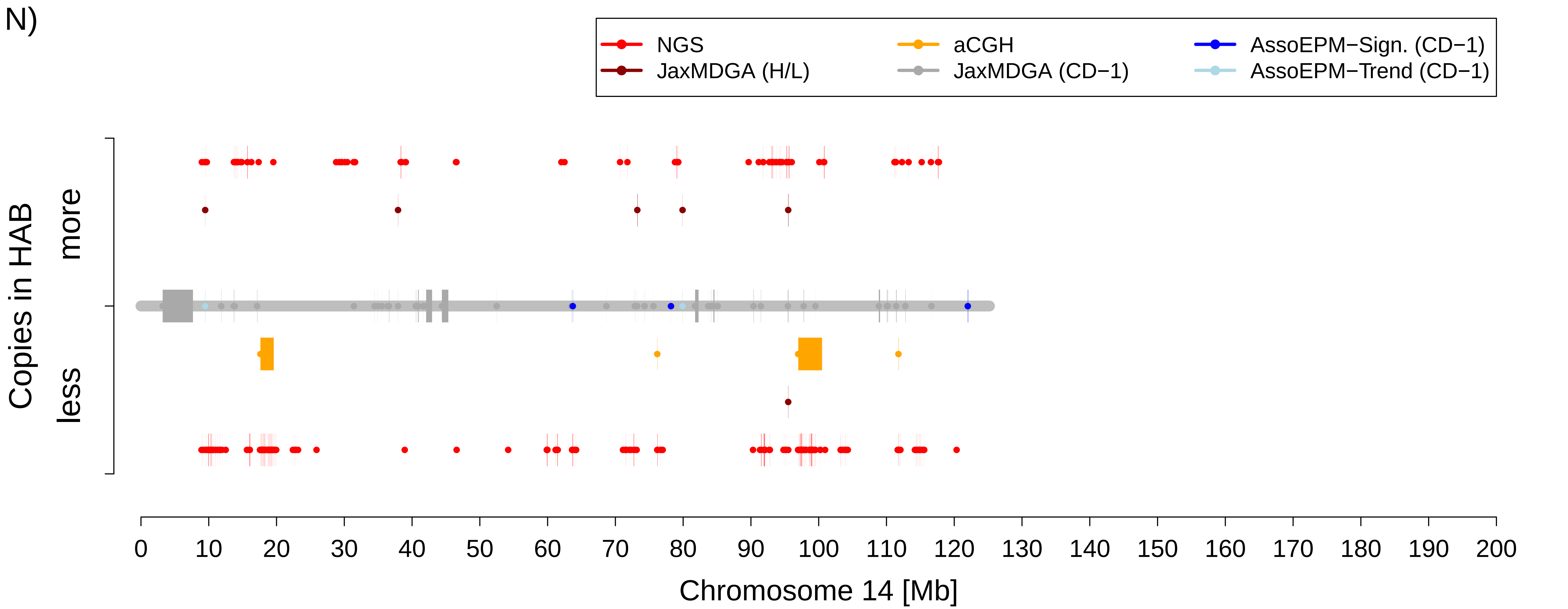
N)
NGS
JaxMDGA (H/L)
aCGH
JaxMDGA (CD−1)
AssoEPM−Sign. (CD−1)
AssoEPM−Trend (CD−1)
Copies in HAB
more
less
0 10 20 30 40 50 60 70 80 90 100 110 120 130 140 150 160 170 180 190 200
Chromosome 14 [Mb]

O)

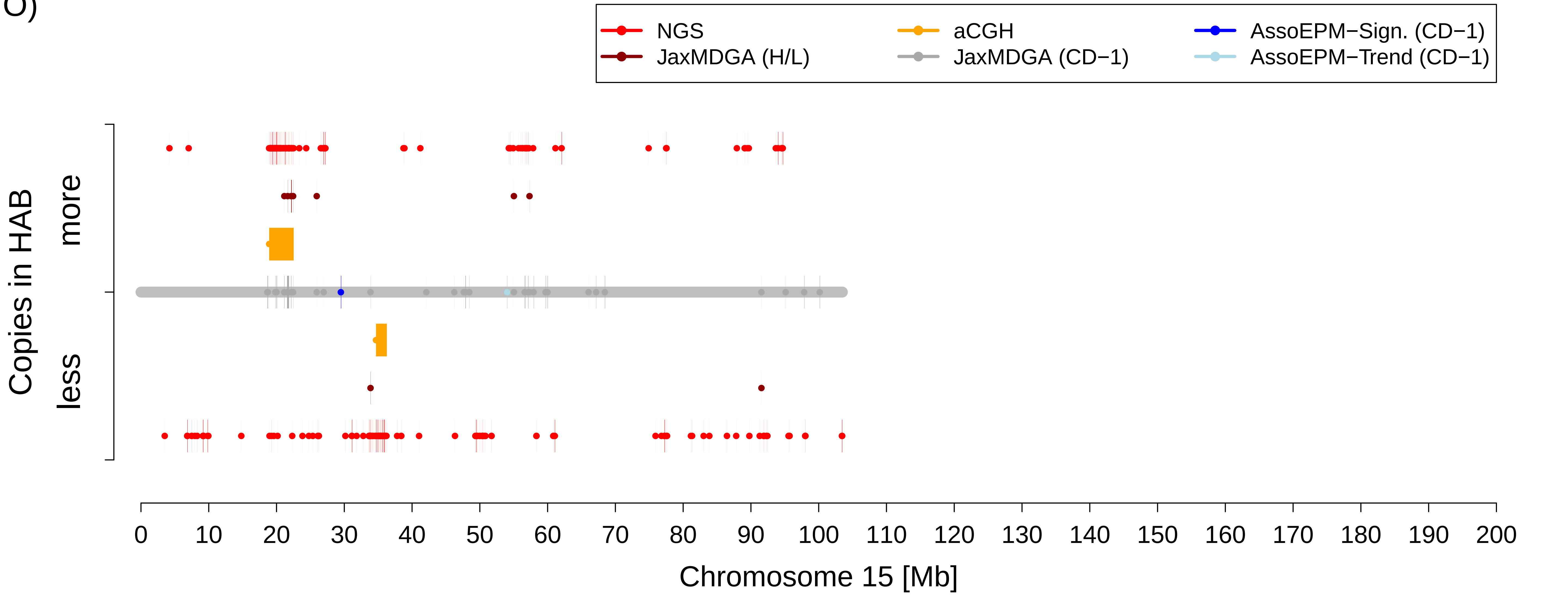

NGS
JaxMDGA (H/L)
aCGH
JaxMDGA (CD−1)
AssoEPM−Sign. (CD−1)
AssoEPM−Trend (CD−1)
Copies in HAB
more
less
0
10
20
30
40
50
60
70
80
90
100
110
120
130
140
150
160
170
180
190
200
Chromosome 15 [Mb]

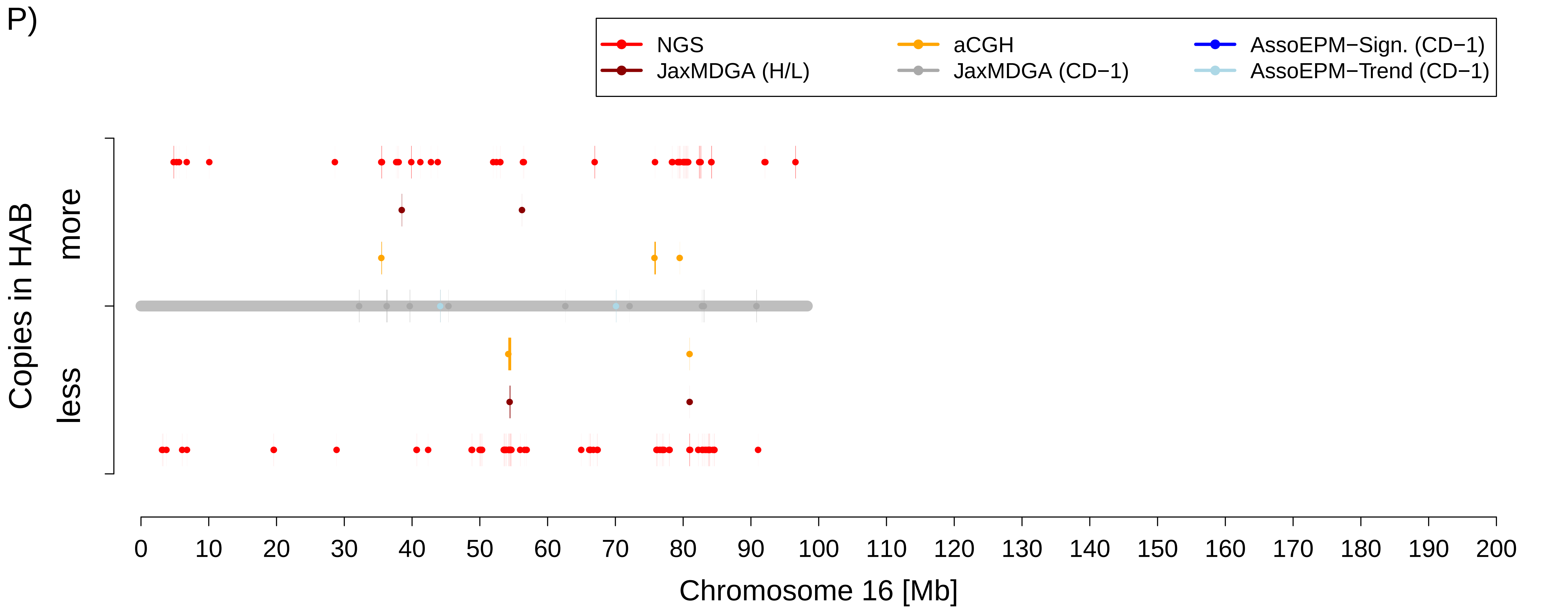

P)
NGS
JaxMDGA (H/L)
aCGH
JaxMDGA (CD−1)
AssoEPM−Sign. (CD−1)
AssoEPM−Trend (CD−1)
Copies in HAB
more
less
0
10
20
30
40
50
60
70
80
90
100
110
120
130
140
150
160
170
180
190
200
Chromosome 16 [Mb]

Q)

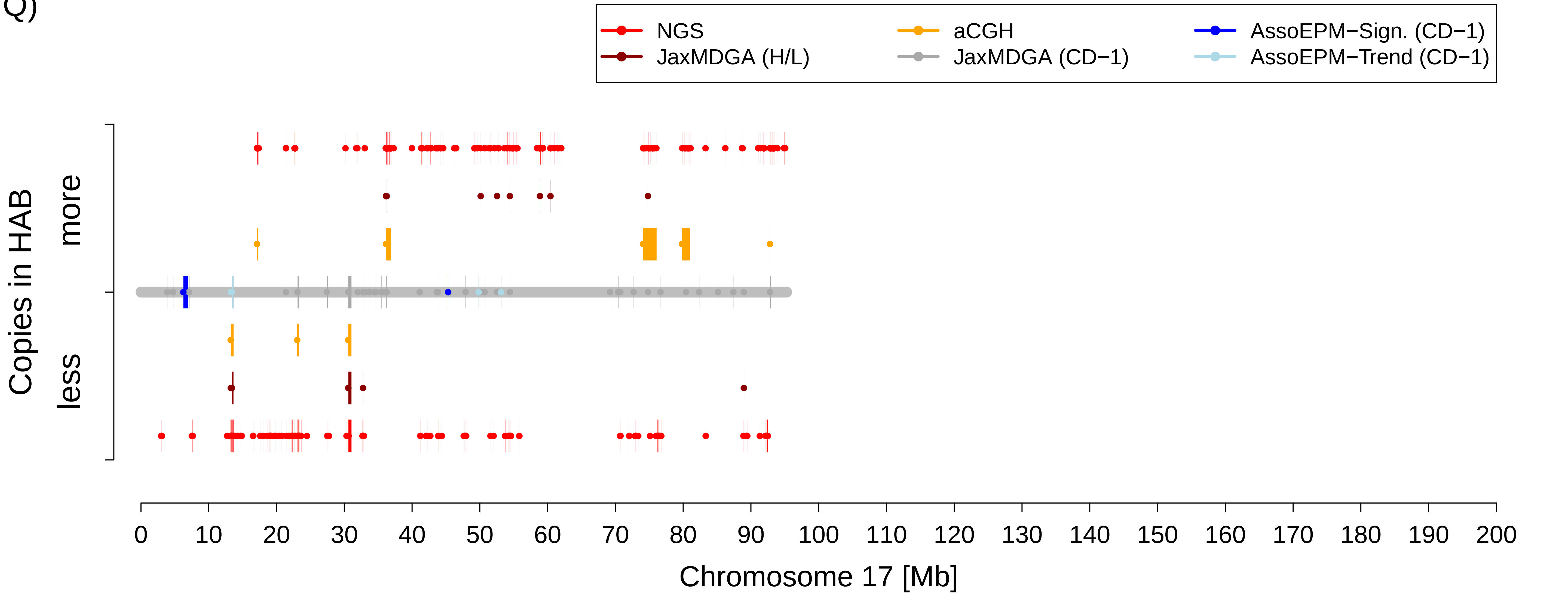

NGS
JaxMDGA (H/L)
aCGH
JaxMDGA (CD−1)
AssoEPM−Sign. (CD−1)
AssoEPM−Trend (CD−1)
Copies in HAB
more
less
0
10
20
30
40
50
60
70
80
90
100
110
120
130
140
150
160
170
180
190
200
Chromosome 17 [Mb]

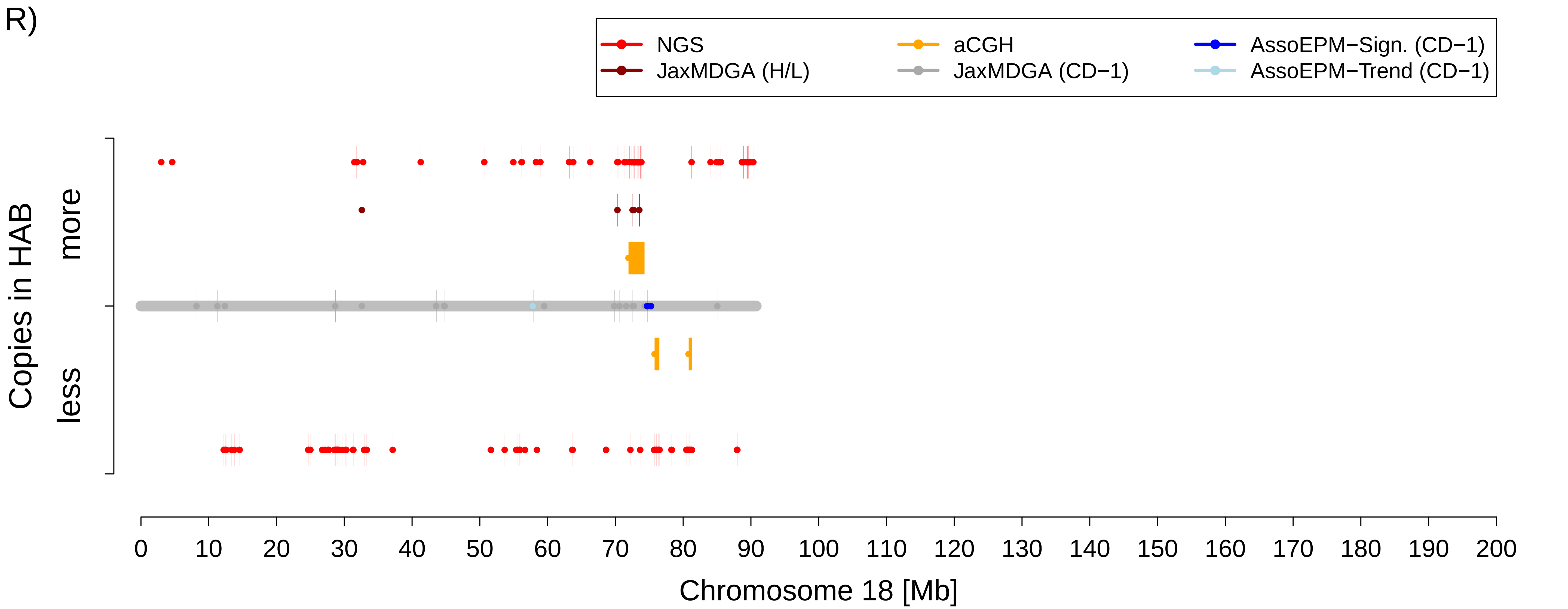

R)
NGS
JaxMDGA (H/L)
aCGH
JaxMDGA (CD−1)
AssoEPM−Sign. (CD−1)
AssoEPM−Trend (CD−1)
Copies in HAB
more
less
0
10
20
30
40
50
60
70
80
90
100
110
120
130
140
150
160
170
180
190
200
Chromosome 18 [Mb]

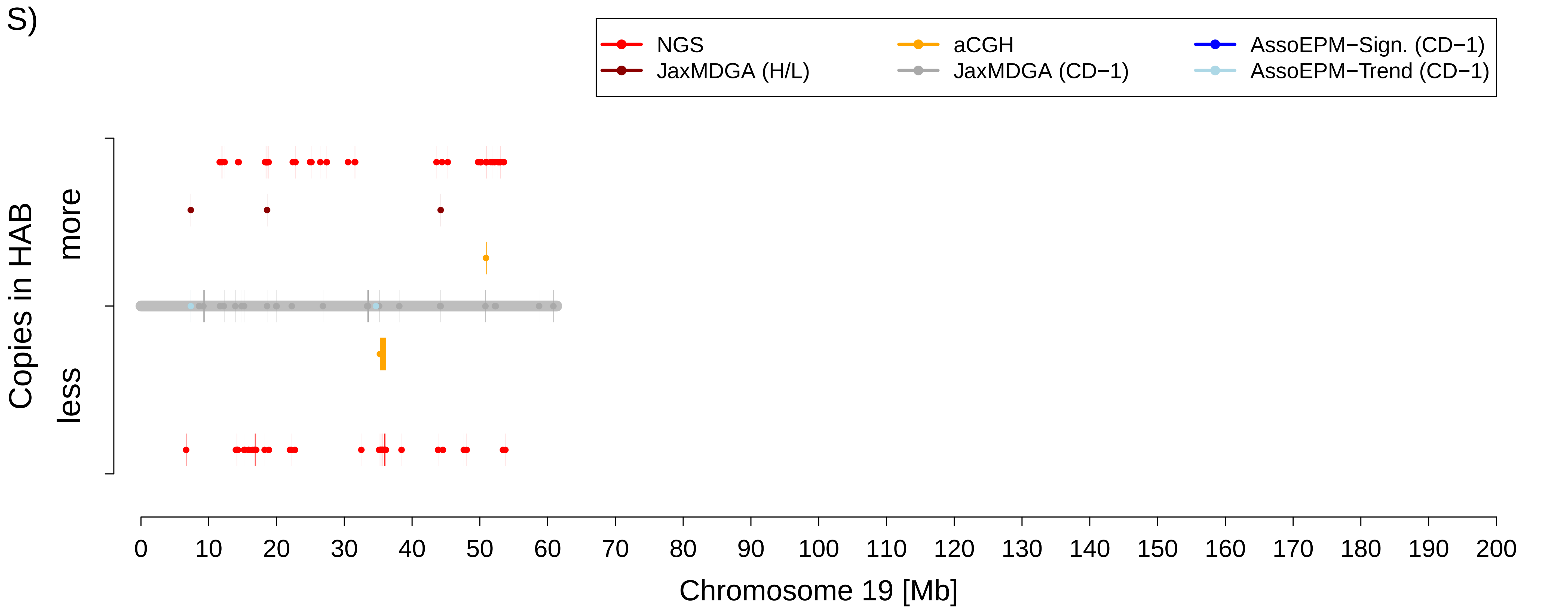
S)
NGS
JaxMDGA (H/L)
aCGH
JaxMDGA (CD−1)
AssoEPM−Sign. (CD−1)
AssoEPM−Trend (CD−1)
Copies in HAB
more
less
0 10 20 30 40 50 60 70 80 90 100 110 120 130 140 150 160 170 180 190 200
Chromosome 19 [Mb]

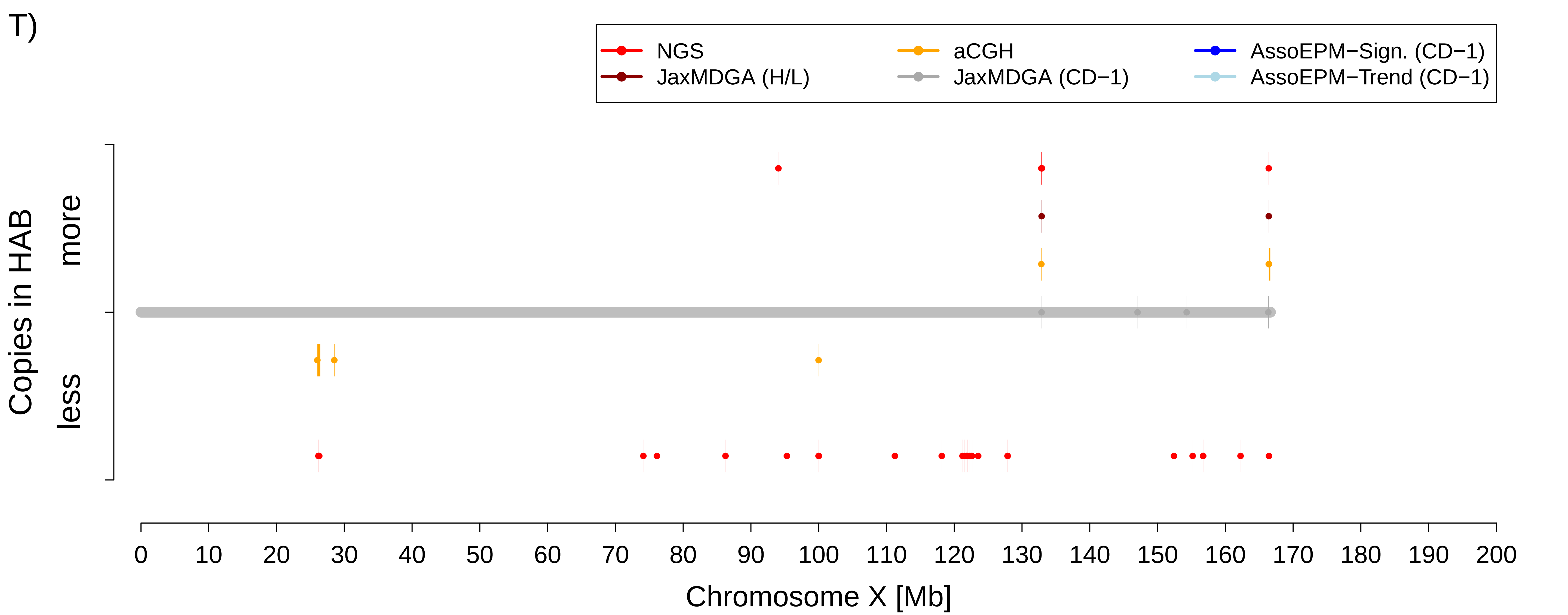

T)
NGS
JaxMDGA (H/L)
aCGH
JaxMDGA (CD−1)
AssoEPM−Sign. (CD−1)
AssoEPM−Trend (CD−1)
Copies in HAB
more
less
0
10
20
30
40
50
60
70
80
90
100
110
120
130
140
150
160
170
180
190
200
Chromosome X [Mb]

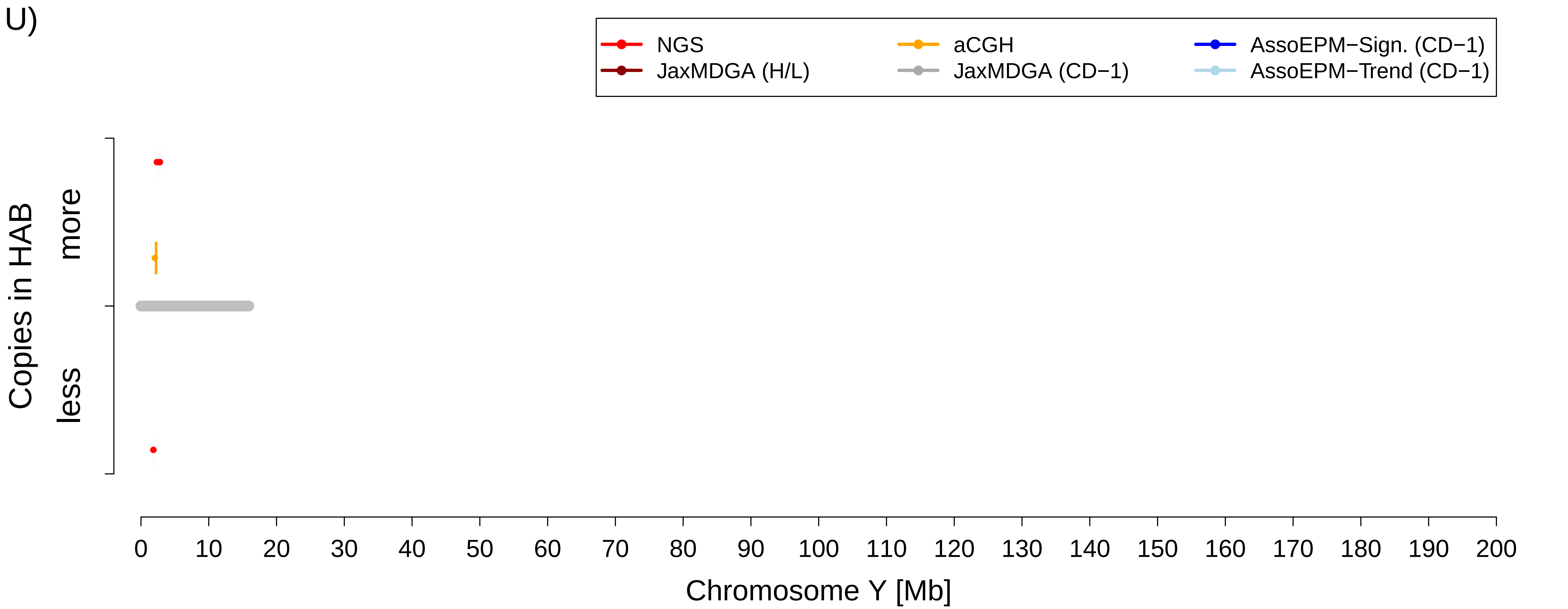

U)
NGS
JaxMDGA (H/L)
aCGH
JaxMDGA (CD−1)
AssoEPM−Sign. (CD−1)
AssoEPM−Trend (CD−1)
Copies in HAB
more
less
0
10
20
30
40
50
60
70
80
90
100
110
120
130
140
150
160
170
180
190
200
Chromosome Y [Mb]
