# Supplementary material for: Connecting Anxiety and Genomic Copy Number Variation: A Genome-Wide Analysis in CD-1 Mice
Source: PLoS One. 2015 May 26;10(5):e0128465. doi: 10.1371/journal.pone.0128465 (PMC4444327; doi:10.1371/journal.pone.0128465)
Supplement: S1 Table — The table is sorted by chromosome. Columns show (left to right): chromosome, gene represented by primer, primer orientation, primer sequence (5’ to 3’), melting temperature and size of the resulting PCR product. (DOC) [file pone.0128465.s007.doc]

**Table S1. Information on primers used for qPCR. The table is sorted by chromosome. Columns show (left to right): chromosome, gene represented by primer, primer orientation, primer sequence (5’ to 3’), melting temperature and size of the resulting PCR product.**

| **Chr.** | **Gene symbol** | **Orientation** | **Primer sequence 5'-->3'** | **T_m_ [°C]** | **Prod. size [bp]** |
| --- | --- | --- | --- | --- | --- |
| 1 | Rgs16 | forward | GGC TCA CCA CAT CTT TGA CG | 59.2 | 110 bp |
|  |  | *reverse* | *TGG TAG TGG CAG CTT GTA GG* | *59.39* |  |
| 2 | B2mg* | forward | CTA TAT CCT GGC TCA CAC TG | 54.74 | 130 bp |
|  |  | *reverse* | *CAT CAT GAT GCT TGA TCA CA* | *53.75* |  |
| 5 | Polr2b* | forward | CAA GAC AAG GAT CAT ATC TGA TGG | 56.95 | 157 bp |
|  |  | *reverse* | *AGA GTT TAG ACG ACG CAG GTG* | *60.07* |  |
| 9 | Glb1 | forward | GTT CTC CGG TCT TCT GAC CC | 59.75 | 85 bp |
|  |  | *reverse* | *AGA GCA GGG GCT TCA TCT TG* | *59.74* |  |
| 11 | Epn2 | forward | CTA GCC TCC CAC CCT AAT GG | 58.94 | 130 bp |
|  |  | *reverse* | *GCT CCT CTT CTC CGC TTG TC* | *60.46* |  |
| 11 | Pdk2 | forward | GAG ATG ACC CCG TCT CCA AC | 59.82 | 120 bp |
|  |  | *reverse* | *GGT TGG TGC TGC CAT CAA AG* | *60.04* |  |
| 12 | Rhoj | forward | ACG CCT TCC CAG AGG AAT AC | 59.17 | 81 bp |
|  |  | *reverse* | *GCA AGT GCT GCT TGC CTC* | *59.74* |  |
| 17 | Alk | forward | GGC AAG CCT GTG ATT TCC AC | 59.76 | 128 bp |
|  |  | *reverse* | *GAG TGG ACT TTG GGT CCA GC* | *60.61* |  |
| 17 | Glo1 | forward | GGA TTG CCG TTC CTG ATG TC | 60.04 | 123 bp |
|  |  | *reverse* | *AGC CGT CAG GGT CTT GAA TG* | *58.98* |  |
| 17 | Slc30a6 | forward | TGG ACC CTT GGA TTT GGC TC | 59.96 | 153 bp |
|  |  | *reverse* | *CCG AAT CCA GTC GTC CTT GA* | *59.47* |  |
| 19 | Gnaq | forward | CAG GAG TGC TAC GAC AGA CG | 60.18 | 82 bp |
|  |  | *reverse* | *CGG CTA CAC GGT CCA AGT C* | *60.45* |  |

* = Housekeeper gene
