# Supplementary material for: Connecting Anxiety and Genomic Copy Number Variation: A Genome-Wide Analysis in CD-1 Mice
Source: PLoS One. 2015 May 26;10(5):e0128465. doi: 10.1371/journal.pone.0128465 (PMC4444327; doi:10.1371/journal.pone.0128465)
Supplement: S3 Table — Upper part: counts of respective CNVs found in HAB/LAB mice. Numbers in parentheses indicate contradictory findings of distinct methods, that is, a copy number loss found by one method and a gain found by at least one other method. Line “overlap both others”shows the number of CNVs defined by the respective detection method that overlap with any other CNV detected by the other two methods. Part below: size of respective CNVs in basepairs (bp). (DOC) [file pone.0128465.s009.doc]

**Table S3. Comparison of CNV detection methods.** *Upper part:* counts of respective CNVs found in HAB/LAB mice. Numbers in parentheses indicate contradictory findings of distinct methods, that is, a copy number loss found by one method and a gain found by at least one other method. Line “overlap both others“ shows the number of CNVs defined by the respective detection method that overlap with any other CNV detected by the other two methods. *Part below:* size of respective CNVs in basepairs (bp).

|  | **aCGH** | **JaxMDGA** | **NGS** |  |
| --- | --- | --- | --- | --- |
| **No. of CNVs… (total)** | 98 | 180 | 5,851 |  |
| **… showing gain in HAB** | 49 | 118 | 2,362 |  |
| **… showing loss in HAB** | 49 | 62 | 3,489 |  |
| **Overlap aCGH (No. CNVs)** | - | 76 (3) | 1,821 (53) |  |
| **Overlap JaxMDGA (No. CNVs)** | 42 (3) | - | 293 (1) |  |
| **Overlap NGS (No. CNVs)** | 97 (22) | 86 (1) | - |  |
| **Overlap both others (No. CNVs)** | 34 (1) | 54 (0) | 221 (1) |  |
|  |  |  |  |  |
| **Total size of CNVs [bp]** | 97,264,699 | 14,731,644 | 27,233,200 |  |
| **Mean size of CNVs [bp]** | 992,497 | 81,842 | 4,654 |  |
| **Median size of CNVs [bp]** | 544,637 | 8,906 | 2,400 |  |
| ***Overlap in all methods [bp]*** | ***4,824,789*** | | |  |
