# Supplementary material for: Connecting Anxiety and Genomic Copy Number Variation: A Genome-Wide Analysis in CD-1 Mice
Source: PLoS One. 2015 May 26;10(5):e0128465. doi: 10.1371/journal.pone.0128465 (PMC4444327; doi:10.1371/journal.pone.0128465)
Supplement: S7 Table — The first part shows the relative expression rate with standard error (SEM) and p-value (calculated by Mann-Whitney-U test) for CeA and BLA, the second part for PVN and Cg. A p-value < 0.05 (bold letters) indicates a significant difference in gene expression between HAB and LAB mice; a p-value < 0.1 (bold and italic letters) indicates a trend. (DOC) [file pone.0128465.s013.doc]

**Table S7. Expression differences of genes tested in qPCR.** The first part shows the relative expression rate with standard error (SEM) and *p*-value (calculated by Mann-Whitney-U test) for CeA and BLA, the second part for PVN and Cg. A *p*-value < 0.05 (bold letters) indicates a significant difference in gene expression between HAB and LAB mice; a *p*-value < 0.1 (bold and italic letters) indicates a trend.

| **Gene**  **symbol** | **CeA**  **relEx HAB**  **+- SEM** | **CeA**  **relEx LAB**  **+- SEM** | **CeA**  **pVal** |  | **BLA**  **relEx HAB**  **+- SEM** | **BLA**  **relEx LAB**  **+- SEM** | **BLA**  **pVal** |
| --- | --- | --- | --- | --- | --- | --- | --- |
| ***^1^Alk*** | 1.0 ± 0.09 | 0.91 ± 0.08 | 0.6203 |  | 1.0 ± 0.12 | 1.24 ± 0.17 | 0.3637* |
| ***^2^Epn2*** | 1.0 ± 0.07 | 0.72 ± 0.08 | **0.0132*** |  | 1.0 ± 0.06 | 0.88 ± 0.03 | 0.1864* |
| ***^3^Glb1*** | 1.0 ± 0.27 | 0.89 ± 0.12 | 0.4574* |  | 1.0 ± 0.14 | 1.78 ± 0.22 | **0.0064** |
| ***^4^Glo1*** | 1.0 ± 0.06 | 2.79 ± 0.20 | **0.0003*** |  | 1.0 ± 0.06 | 3.07 ± 0.22 | **0.0003***^T^ |
| ***^5^Gnaq*** | 1.0 ± 0.08 | 0.95 ± 0.14 | 0.409* |  | 1.0 ± 0.08 | 1.12 ± 0.22 | 0.8688* |
| ***^6^Pdk2*** | 1.0 ± 0.09 | 0.78 ± 0.03 | **0.0318** |  | 1.0 ± 0.12 | 0.93 ± 0.12 | 0.6203 |
| ***^7^Rgs16*** | 1.0 ± 0.16 | 0.84 ± 0.15 | 0.409* |  | 1.0 ± 0.09 | 0.85 ± 0.15 | 0.1864* |
| ***^8^Rhoj*** | 1.0 ± 0.10 | 1.18 ± 0.09 | 0.1604* |  | 1.0 ± 0.09 | 1.31 ± 0.14 | **0.0475** |
| ***^9^Slc30a6*** | 1.0 ± 0.18 | 0.64 ± 0.06 | ***0.0575*****^T^* |  | 1.0 ± 0.12 | 1.16 ± 0.10 | 0.2831* |
|  | **PVN**  **relEx HAB**  **+- SEM** | **PVN**  **relEx LAB**  **+- SEM** | **PVN**  **pVal** |  | **Cg**  **relEx HAB**  **+- SEM** | **Cg**  **relEx LAB**  **+- SEM** | **Cg**  **pVal** |
| ***Alk*** | 1.0 ± 0.14 | 1.66 ± 0.81 | 0.7412 |  | 1.0 ± 0.10 | 1.31 ± 0.09 | **0.0132** |
| ***Epn2*** | 1.0 ± 0.07 | 0.89 ± 0.20 | 0.1604* |  | 1.0 ± 0.12 | 0.92 ± 0.07 | 0.8590* |
| ***Glb1*** | 1.0 ± 0.07 | 1.94 ± 0.42 | **0.0166*** |  | 1.0 ± 0.11 | 1.23 ± 0.14 | 0.1167* |
| ***Glo1*** | 1.0 ± 0.11 | 3.25 ± 0.36 | **0.0003*** |  | 1.0 ± 0.15 | 2.65 ± 0.25 | **0.0004*** |
| ***Gnaq*** | 1.0 ± 0.21 | 1.51 ± 0.27 | 0.1167* |  | 1.0 ± 0.16 | 0.45 ± 0.04 | **0.0022*** |
| ***Pdk2*** | 1.0 ± 0.09 | 1.87 ± 1.04 | 0.5089 |  | 1.0 ± 0.17 | 0.91 ± 0.10 | 0.8688 |
| ***Rgs16*** | 1.0 ± 0.12 | 1.08 ± 0.36 | 0.3218* |  | 1.0 ± 0.09 | 0.85 ± 0.12 | 0.1604 |
| ***Rhoj*** | 1.0 ± 0.11 | 1.74 ± 0.61 | **0.039*** |  | 1.0 ± 0.13 | 0.90 ± 0.13 | 0.2477* |
| ***Slc30a6*** | 1.0 ± 0.27 | 1.27 ± 0.54 | 0.6797* |  | 1.0 ± 0.19 | 0.95 ± 0.10 | 0.8688* |

^1^MGI:103305; ^2^MGI:1333766; ^3^MGI:88151; ^4^MGI:95742; ^5^MGI:95776; ^6^MGI:1343087; ^7^MGI:108407; ^8^MGI:1931551; ^9^MGI:2386741; * = confirmation of microarray result by at least one microarray probe; *T = potential confirmation of microarray result, with one of the methods showing a trend
