# Supplementary material for: Connecting Anxiety and Genomic Copy Number Variation: A Genome-Wide Analysis in CD-1 Mice
Source: PLoS One. 2015 May 26;10(5):e0128465. doi: 10.1371/journal.pone.0128465 (PMC4444327; doi:10.1371/journal.pone.0128465)

**Supporting Material and Methods**

***Correlation analysis of CNV and expression data***

The calculation of the two proportion *Z*-test was executed in R as described below:

n1 = number of genes not showing significant expression difference

(all: 8607, BLA: 8881, CeA: 8725, Cg: 8759, PVN: 8721)

n2 = number of genes differentially expressed between HAB and LAB mice

(all: 374, BLA: 100, CeA: 256, Cg: 222, PVN: 260)

cnvDiff = number of differentially expressed genes that include or are included in CNV regions which were detected by all three methods, i.e. aCGH, JaxMDGA and NGS

(all: 22, BLA: 9, CeA: 13, Cg: 17, PVN: 15)

cnvNodiff = number of genes not differentially expressed that include or are included in CNV regions which were detected by all three methods, i.e. aCGH, JaxMDGA and NGS

(all: 46, BLA: 59, CeA: 55, Cg: 51, PVN: 53)

$$pro1=\frac{cnvNodiff}{n1}$$

$$pro2=\frac{cnvDiff}{n2}$$

$$pro3=\frac{cnvDiff+cnvNodiff}{n1+n2}$$

$$SE=\sqrt{pro3\times\left(1-pro3\right)\times\left(\frac{1}{n1}+\frac{1}{n2}\right)}$$

$$z=\frac{pro1-pro2}{SE}$$

$$p-value=2\times pnorm\left(-abs\left(z\right)\right)$$

***Explanation, why CNV calling was improved by comparing all CD-1 samples to each other:***

Based on the applied algorithm for CNV calling, it might happen that an existing CNV would not be recognized as such if samples were compared to one single reference solely. In the figure below you see an example of a genomic region. Normalized fluorescence intensities of single probes within that region are depicted as single dots in green (reference sample, Ref), red (sample 1, S1) and blue (sample 2, S2).

Comparisons of sample intensities to the reference intensities might not show significant differences to be recognized by the applied algorithm, indicated by the orange crosses, and hence a potential CNV (orange lines) would be missed. In contrast, a direct comparison of sample 1 with sample 2 intensities could pass the significance threshold (orange star).


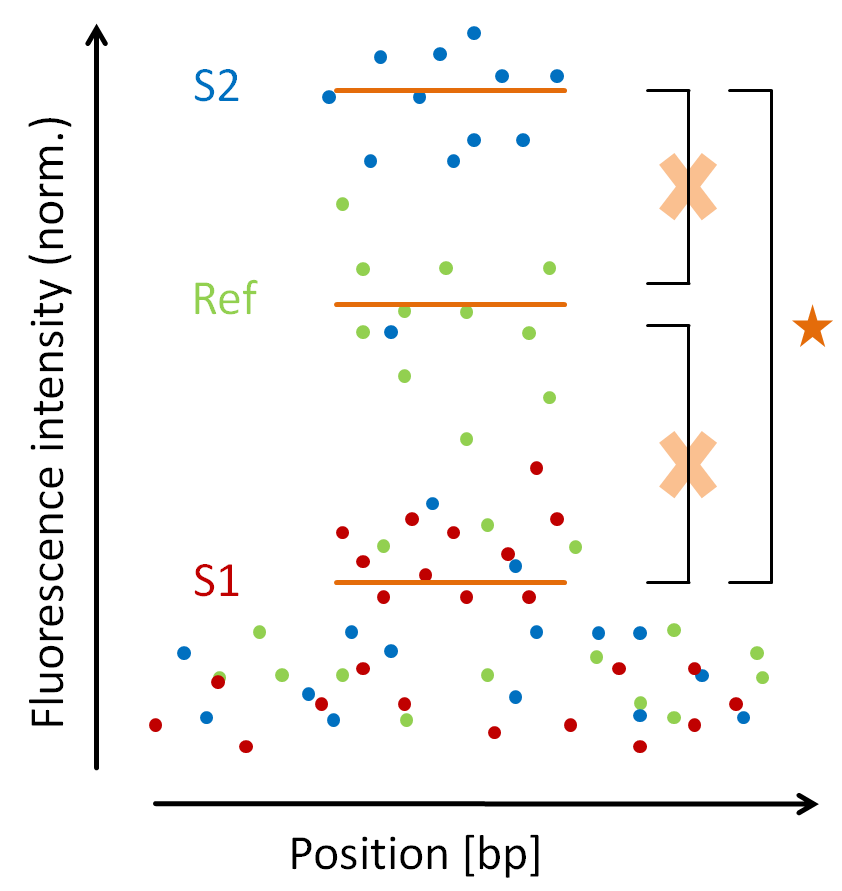

Supplement: S1 Text — (DOC) [file pone.0128465.s015.doc]
